# Cochlear Implant Outcomes and Genetic Mutations in Children with Ear and Brain Anomalies

**DOI:** 10.1155/2015/696281

**Published:** 2015-07-05

**Authors:** Micol Busi, Monica Rosignoli, Alessandro Castiglione, Federica Minazzi, Patrizia Trevisi, Claudia Aimoni, Ferdinando Calzolari, Enrico Granieri, Alessandro Martini

**Affiliations:** ^1^Department of Medical & Surgical Disciplines of Communication and Behavior, University Hospital of Ferrara, Via Fossato di Mortara 64/A, 44121 Ferrara, Italy; ^2^ENT & Audiology Department, University Hospital of Ferrara, Via Aldo Moro 8, 44124 Ferrara, Italy; ^3^Department of Neurosciences, Complex Operative Unit of Otorhinolaryngology and Otosurgery, University Hospital of Padua, Via Giustiniani 2, 35128 Padua, Italy; ^4^Neuroradiology Service, University Hospital of Udine, Piazzale Santa Maria della Misericordia 15, 33100 Udine, Italy; ^5^Neurological Clinic, University Hospital of Ferrara, Via Aldo Moro 8, 44124 Ferrara, Italy

## Abstract

*Background*. Specific clinical conditions could compromise cochlear implantation outcomes and drastically reduce the chance of an acceptable development of perceptual and linguistic capabilities. These conditions should certainly include the presence of inner ear malformations or brain abnormalities. The aims of this work were to study the diagnostic value of high resolution computed tomography (HRCT) and magnetic resonance imaging (MRI) in children with sensorineural hearing loss who were candidates for cochlear implants and to analyse the anatomic abnormalities of the ear and brain in patients who underwent cochlear implantation. We also analysed the effects of ear malformations and brain anomalies on the CI outcomes, speculating on their potential role in the management of language developmental disorders. *Methods*. The present study is a retrospective observational review of cochlear implant outcomes among hearing-impaired children who presented ear and/or brain anomalies at neuroimaging investigations with MRI and HRCT. Furthermore, genetic results from molecular genetic investigations (*GJB2/GJB6* and, additionally, in selected cases, *SLC26A4* or mitochondrial-DNA mutations) on this study group were herein described. Longitudinal and cross-sectional analysis was conducted using statistical tests. *Results*. Between January 1, 1996 and April 1, 2012, at the ENT-Audiology Department of the University Hospital of Ferrara, 620 cochlear implantations were performed. There were 426 implanted children at the time of the present study (who were <18 years). Among these, 143 patients (64 females and 79 males) presented ear and/or brain anomalies/lesions/malformations at neuroimaging investigations with MRI and HRCT. The age of the main study group (143 implanted children) ranged from 9 months and 16 years (average = 4.4; median = 3.0). *Conclusions*. Good outcomes with cochlear implants are possible in patients who present with inner ear or brain abnormalities, even if central nervous system anomalies represent a negative prognostic factor that is made worse by the concomitant presence of cochlear malformations. Common cavity and stenosis of the internal auditory canal (less than 2 mm) are negative prognostic factors even if brain lesions are absent.

## 1. Background

Cochlear implantation (CI) is a significant surgical innovation of the 20th century and is the first artificial sensory organ used in clinical practice. Currently, CI is an effective medical procedure. Nonetheless, there remain certain controversial issues from economic, clinical, and ethical point of view, especially in specific clinical conditions that could compromise the CI outcome and drastically reduce the chance of an acceptable development of perceptual and linguistic capabilities [1]. The CI has been devised to allow full access to verbal communication through the perception of phonetic hallmarks. The success of this method is then given in general by the achievement of verbal communication performance by improving the skills of verbal perception to become comparable to people with normal hearing [2]. In the paediatric population, in children with profound hearing loss, which is unsuitable for obtaining significant results with traditional hearing aids, CI (if performed early) allows the optimal development of auditory and linguistic abilities, which drives toward adequate communication and intellectual development [2, 3]. Children with congenital profound hearing loss accumulate disadvantages over time in language skills and certain learning areas, which can lead to permanent limitations of personal skills at a later age. Because of the auditory habilitation/rehabilitation training with a cochlear implant, most of these patients can reach a complete disability “compensation” [2, 4]. Several studies have reported the results of the development of auditory perceptual and expressive verbal abilities in children with pre-, peri-, and postlingual deafness [3, 4]. Clinical experiences across the world have also shown that, among children with preverbal onset hearing loss, there is a critical period for the development of language skills, presumably due to the underlying neuronal plasticity, and learning would be strictly dependent on the presence of an adequate auditory input, which explains the need for early intervention to prevent the occurrence of a delay in language development and perceptive or expressive skills [5, 6].

The performance of the patients who received CIs varies significantly as a consequence of a substantial number of audiological and extra-audiological factors (age of hearing loss onset, duration of auditory deprivation, auditory function residuals, presence of associated disability and comorbidity, language skills at the time of the CI, duration of CI use, the presence of certain malformations of the inner ear, socioeconomic status, and familial environment) [7–9]. Even if a unique prediction of the results after CI is not yet available, mostly because of the extreme heterogeneity in the aetiology among profound hearing-impaired patients, there are many prognostic factors that can contribute to the audiological assessment [4, 10–12]. Among these, we emphasise the importance of inner malformations and brain anomalies.

Because cochlear implantation is an invasive and expensive surgical procedure, the identification of predictive factors is one of the most important goals; knowledge of the predictive factors can help to guide rehabilitation programs that are tailored to meet the expectations of clinicians, teachers, and parents [9]. The perception of verbal sounds is an important starting point to activate the processes of linguistic acquisition. The development of language depends on auditory skills and maturation of cortical functions (memory, attention, and intellectual abilities) [13, 14]. The linguistic processes typically follow the perceptive processes with a variable latency, which appears to be related to the age of the patients at the time of surgery [15, 16]. Specifically, children implanted in a very early age (8–12 months) experience linguistic evolution with a speed that is higher than that of normal hearing children of the same age, probably because of using lines of development in various linguistic domains that are different from the usual capabilities of normal hearing children [14, 15]. In other words, congenitally deaf patients develop many abilities to reach an adequate communication condition (lip-reading, visual reinforcement) that, when auditory function has recovered, work in synergy with auditory inputs, enhancing perceptual and visual skills; an analogous process is similar in visually impaired patients who develop, more than normal, auditory and olfactory skills [2]. On the other hand, it cannot be excluded that the normal auditory input is more detailed and complex and, thus, that it takes more time to develop and integrate superior central functions. In contrast, cochlear implant stimuli are simpler; thus, they do not need complex integration in the corpus callosum or cortical areas [17].

With the advances in molecular genetics over the past 20 years, our understanding of the pathogenesis of sensorineural hearing loss has greatly increased [18–21]. The most common mutations that are responsible for hearing loss involve the* GJB2* gene;* SLC26A4* mutations are the second cause of genetic hearing loss and the first among syndromic deafness. The* SLC26A4* (PDS) gene mutations result in abnormalities of the endolymphatic system, which lead to the dilation of the vestibular aqueduct as seen in Pendred syndrome. Several studies have shown that patients who have mutations of* GJB2* (or Cx26) (OMIM ∗ 121011) usually have excellent perception of speech and an optimal language development after the cochlear implant [22–24]. Additionally, it has been reported that* GJB2* mutations are not usually accompanied by macroscopic inner ear malformations [19]. Nevertheless, there is no evidence that genetic mutations or the interaction of a genetic diagnosis with other prognostic factors (such as abnormalities of the ear and brain) can predict CI outcomes.

Preoperative neuroimaging is mandatory in cochlear implant candidates for diagnostic and surgical purposes. This step usually includes an MRI as well as a high-resolution computed tomography (HRCT) of the temporal bone. MRI should be performed with contrast (gadolinium), unless otherwise noted or unless the test is in children who are not believed to have lesions that require contrast to be diagnosed. HRCT of the temporal bone does not require iodine contrast. Note that magnetic resonance imaging (MRI) is relatively contraindicated after cochlear implantation or it is arguably possible. Various experimental studies have shown that MRI scans can safely be performed with the CI in place [25]. This arrangement does not imply that it is generally safe to perform MRI in CI patients, because the type of implant, fixation method, and MRI units and sequences could vary. Even if it can be performed safely, the distortion that is caused by the implanted magnet will cause suboptimal interpretations. For the aforementioned reasons, cochlear implantation can be contraindicated in patients who need periodic follow-up with MRI [25].

At our clinic, preoperative radiological imaging of cochlear implant candidates includes both HRCT and MRI of the temporal bone during the same session, during anaesthesia, if required, which usually occurs in children. In addition to the MRI of the inner ear, we also perform brain and brainstem MRI scans. These scans enable us to exclude any incidental brain abnormalities that can contraindicate CI surgery. MRI is the best diagnostic tool for detecting malformations such as cochlear nerve hypoplasia or aplasia, and it is the best screening tool for early cochlear ossification following bacterial meningitis [26, 27]. HRCT provides better images and definition of the facial nerve canal, middle ear, and otic capsule [28]. Central nervous system findings have been reported in 20–40% of the patients [29–32]. Some of these findings could result in neurodevelopmental delay and could negatively impact the outcome of cochlear implantation [30]. Nonetheless, increased experience in cochlear implantation has led to more children with abnormal cochleovestibular anatomy being considered as candidates [4, 11]. According to the literature, approximately 20% of the children who have sensorineural hearing loss have associated radiological anomalies of the temporal bone [33–36]. These temporal bone anomalies are accompanied by a wide range of hearing acuity, varying degrees of progression of hearing loss, and the presence or absence of related nonotological anomalies [33].

In general, “cochlear implantation is a relatively safe procedure with a low complication rate that ranges from 6% to 20%. Major complications are those that are life threatening or require surgery, whereas minor complications are those that can be medically treated. The inner ear malformations can increase the risk of meningitis, cerebrospinal fluid leakage, and facial nerve palsy” [37–41]. We should note that the rate of postoperative complications was higher in patients with anomalous inner ears than in patients with normal inner ears; most of them were minor and could be managed conservatively [41]. Nevertheless, the functional results reached by these children (perceptual and linguistic performance) are still poorly described and have not been predictable. Case studies have limited conclusions because of the high interindividual variability. For these reasons, it is not yet possible to draw clear guidance from the literature on which to base the selection of candidates [12, 13, 41]. The malformations in fact allow the correct insertion of a number of electrodes that are usually sufficient, and the patterns of neural responses are adequate to accomplish the recognition of an open set words. However, specific conditions that prevent a correct coupling between the electrode array and the cochlear nerve, even if the latter is present, such as a common cavity, are usually characterised by a poor outcome, unless very specific surgical strategies are enacted.

**Figure 1:**
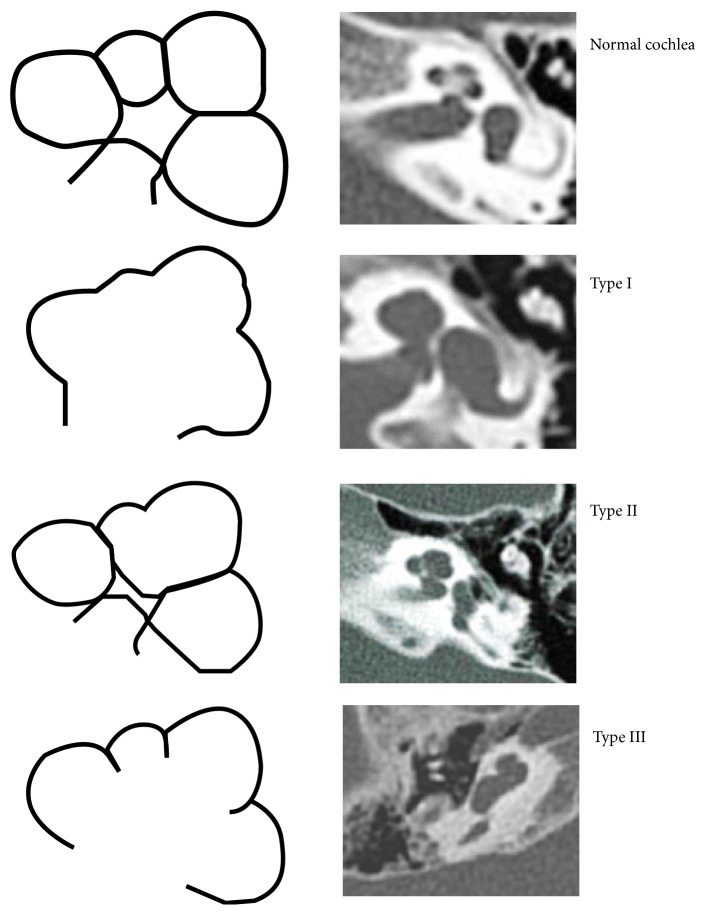
Incomplete partition types [45].

**Figure 2:**
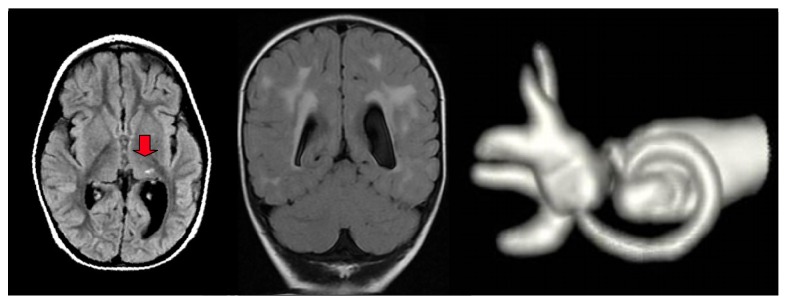
MRI scans, after effects of CMV meningoencephalitis, with patchy lesions of the white matter (red arrow) and dilation of the left lateral ventricle. 3D MR image of a case of semicircular canal occlusion, complication of CMV meningoencephalitis.

**Figure 3:**
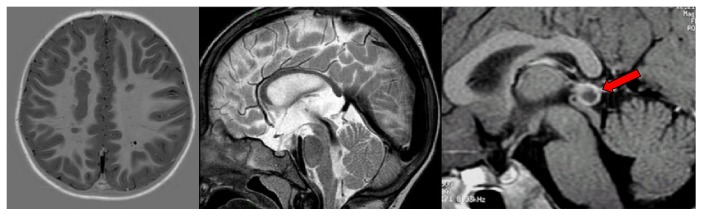
MRI scans: gray matter heterotopia, hypoplasia of the corpus callosum. Red arrow: pineal cyst.

**Figure 4:**
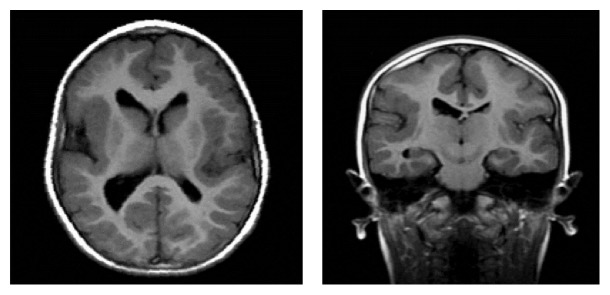
MRI scans. Cortical dysplasia.

**Figure 5:**
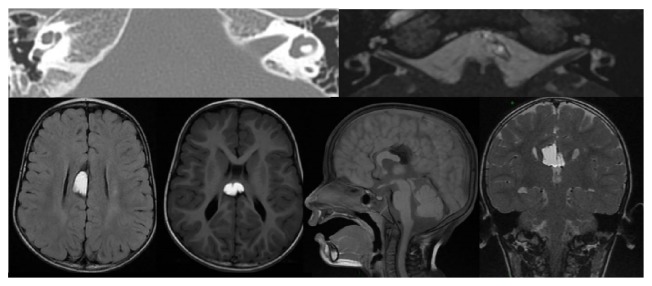
CT and MRI scans. Hypoplastic lateral semicircular canal; lipoma of the corpus callosum; agenesis of the posterior part and splenium of the corpus callosum; pellucid septum cyst; dilated cisterna magna (or cerebellomedullary cistern).

Approximately 80% of the children who have a congenital hearing loss have no macroscopic abnormalities of the ear, and their hearing loss is assumed to be the result of dysfunctions at a cellular level in the membranous inner ear. The remaining 20% can present inner ear dysplasia, which can be demonstrated on high-quality neuroimaging (HRCT without contrast and MRI, with contrast in adults or in specific cases). The inner ear abnormalities, whether dysplastic or nondysplastic, can be isolated or can be part of a multiorgan syndrome [19]. Developmental malformations that affect the otic capsule result in anomalies of both the membranous and bony labyrinth. The specific timing of the insult during otic capsule development determines the resulting type of malformation along a spectrum of congenital inner ear malformations that can occur when the normal process of development is impacted, even if it is not necessarily understood why this result occurs. The best review of these developmental anomalies is given by Cullen et al. [24] and Heller et al. [25], which is an update on the valuable original work by Jackler et al. [34], and the present study is essentially based on their classification (Figure 1) [42–46]. In clinical practice, CNS lesions are usually represented by neoplasms/neoformations, malformations, vascular/ischemic and gliotic lesions, white-matter disorders, demyelinating disorders, and viral/bacterial infections (meningitis and cytomegalovirus infections) (Figures 2, 3, 4, and 5).

The aims of this work were to study the diagnostic value of HRCT and MRI in children who have sensorineural hearing loss and who were candidates for cochlear implants; we also aim to analyse anatomic abnormalities of the ear and brain in patients who underwent CI. We analysed the effects of ear malformations and brain anomalies on the language development and CI outcomes. Finally, we described the genetic mutations that we found in the study group. A control study group of implanted patients without ear and brain anomalies was obtained (virtually) from clinical data and literature data for statistical purposes [47–50].

## 2. Methods

This study is a retrospective observational review of cochlear implant outcomes among hearing-impaired children who presented ear and/or brain anomalies at neuroimaging investigations with MRI and HRCT. Furthermore, genetic results from molecular genetic investigations (*GJB2*/*GJB6* and, additionally, in selected cases,* SLC26A4* or* mitochondrial-DNA* mutations) on this study group were herein described. Longitudinal and cross-sectional analyses were conducted using statistical tests. To create more homogenic groups and more study-specific findings (e.g., EVA) and to obtain more significant analysis, the main study group was divided into different subgroups, which were named with alphabetic letters.

A control study group was created starting from literature data and randomised selected cases (from our casuistry) of implanted children without neuroradiological findings [50]. A long-term follow-up was performed, which reported that the Geers and Moore score was achieved at 3, 6, 12, 24, and 36 months. Each of the subgroups was compared with the control study group. Furthermore, the subgroups were compared with each other only if the same patients were not present in either. Specific findings were reported as singular cases. A nonparametric test was used for statistical analysis: the Mann-Whitney *U* test. Furthermore, we used the ANOVA test (analysis of variance) for the comparison between patients with monolateral CI and patients with bilateral CI.

### 2.1. Audiological Assessment

Before implantation, all of the children had documented severe to profound or profound sensorineural HL (hearing loss) and failed an appropriate hearing-aid trial. Each patient has been investigated from an audiological point of view using objective tests, such as OAEs (otoacoustic emissions), ABR (auditory brainstem response), and ASSR (auditory steady state response), to estimate the pure-tone threshold and, in selected cases, to perform ECochG (electrocochleography). When possible, the audiological assessment was completed using behavioural and tonal audiometry with and without previously described hearing aids.

Children of 6–36 months of age underwent conditioned orientation reflex (COR) and visual reinforcement audiometry (VRA) tests to investigate the tonal threshold at low frequencies and to assess the effectiveness of the hearing aids. From the age of three to four years, the pure-tone hearing thresholds can be obtained by motivational games that range from peep shows to finger-raising techniques, and at an age of six years, most children can perform formal audiometry the same as that used in adults. The testing is dependent only on the degree of cooperation of the child and the experience of the tester. Microotoscopy, tympanometry, and recording of stapedius reflex thresholds were part of the test procedure. The interpretation and diagnostic validity of stapedius-reflex-threshold testing in children are similar to the testing of adults, but the test might be difficult to perform in very young children.

### 2.2. Imaging Data

High resolution HRCT (high resolution computed tomography) and MRI (magnetic resonance imaging) were conducted in all patients to obtain a radiological examination of the temporal bone and brain. If necessary, children underwent neuroradiological scans during general anaesthesia. HRCT scanning with contiguous 0.3–1 mm thick images through the petrous temporal bone in the axial and direct coronal planes was performed. Ear, brainstem, and encephalon MRI scanning was acquired at 1.5 T and included high resolution axial and coronal T2-weighted imaging axial and coronal T1-weighted imaging, using CISS (constructive interference in steady state) and FIESTA (fast imaging employing steady state acquisition). If contrast was required, then postcontrast T1-weighted images were acquired in all three planes. CISS and FIESTA are a gradient-echo MRI sequence that are used to investigate a wide range of pathologies when routine MRI sequences do not provide the desired anatomic information. MRI brain scanning was also acquired at 1.5 T and included axial T2-weighted imaging, axial fast fluid-inversion recovery sequence (FLAIR) imaging, axial T1-weighted inversion recovery imaging, and, if contrast was required, axial T1-weighted imaging.

The neuroimaging findings of the temporal bone were categorised as

1. cochlear malformations,
2. vestibular and semicircular canal malformations,
3. IAC (internal auditory canal) anomalies,
4. EVA (enlarged vestibular aqueduct).

The vestibular aqueduct is defined as enlarged if its diameter is greater than 1.5 mm at the midpoint. Vestibular and labyrinthine abnormalities included partial SCC aplasia and total SCC aplasia. Cochlear malformations were subsequently divided as follows:

1. cochlear malformations:
  - common cavity deformity,
  - cochlear hypoplasia,
  - incomplete partition type I (IP-I),
  - incomplete partition type II (IP-II) (Mondini deformity),
  - incomplete partition type III (IP-III),
  - basal turn dysplasia.

Mondini malformation is a cochlear anomaly that is characterised by a fusion of the apical and middle turn (only one and a half turns are present out of the normal two and a half turns).

The brain MRI scans of all of the patients were reviewed, and all of the abnormal findings were identified and classified as follows:

1. malformations:
  - aplasia, dysplasia, or hypoplasia,
  - dilatations,
  - Arnold-Chiari malformations;
2. neoformations:
  - neoplasms (benign or malignant),
  - cystic lesions;
3. white matter disorders:
  - leukomalacia,
  - *leukodystrophy*,
  - demyelination;
4. gliotic lesions (including cytomegalovirus infections and ischemic lesions);
5. other abnormalities.

### 2.3. Genetic and Molecular Analysis

Informed consent was obtained from patients and parents according to current national rules and laws. Molecular genetic studies of the* GJB2*,* GJB6*, and* SLC26A4* genes and mitochondrial DNA (mit-DNA) were performed in 77 patients. Genomic DNA was extracted by standard protocols from peripheral blood leukocytes of patients. Direct DNA sequencing of the* GJB2* gene (including analysis of the entire coding region) was performed. PCR amplification of the coding 21 exons, the flanking, and promoter regions of the* SLC26A4* gene was performed using specific primers. Amplification reactions were performed in a final volume of 25 mL containing 100 ng of genomic DNA, 200 mmol/L dNTPs, 10 mmol/L each primer 1.5 mmol/L MgCl_2_, and 1 U of Taq polymerase. After 5 min of denaturation at 94°C, 35 PCR cycles were carried out, each cycle comprising 45 s of denaturation at 94°C, 45 s of annealing at 60°C, and 80 s of extension at 72°C. Direct sequencing of the PCR products on both strands was performed on an ABI PRISM 3130xl sequencer, using the ABI BigDye Terminator v3.1 Cycle Sequencing Kit (Applied Biosystems by Life Technologies).

### 2.4. Speech Perception (Preoperative Assessment and Postoperative Outcomes)

Behavioural measures of speech perception scores [50–54] are routinely completed in all children in our study at follow-up visits and a database of these outcomes is maintained. The database also includes patient demographics (age at implant, gender, and duration of implant use), audiological characteristics (congenital versus progressive loss), and relevant medical history (other medical conditions, such as craniofacial syndromes).

Perceptive abilities are usually classified into 4 types of increasing complexity performances [52, 53]:

1. detection: ability to respond to the presence or absence of a signal;
2. discrimination: ability to distinguish differences or similarities between two stimuli;
3. identification: ability to choose an item from a known set;
4. recognition: ability to repeat or imitate spoken stimuli.

The achieved performance enabled us to include each patient in a specific perceptual category. Geers and Moog proposed perceptive classification with six categories, which are based on performances that have been analysed with sets of specific tests [14].

Geers and Moog perception was used for the present study. A comparison of specific speech perception tests was conducted between hearing impaired children with normal anatomy (called “well babies”) and those who were affected by cochleovestibular and brain abnormalities. An excellent tool for monitoring progress in young children is the Clinical Red Flag Procedure [50], which is a matrix of auditory benchmarks that has been established for identifying children who are progressing at a slower-than-expected rate. These benchmarks are based on research and clinical findings that document the listening skills that are achieved by the average CI child during the first year of device use. Three different groups of CI children reflect different preimplant characteristics and show different patterns of skill achievement [50].

It appears evident that in the “well babies,” the perceptual expected results after 3 months of use of the CI essentially comprise the detection of voice and first discrimination abilities until the recognition of words and phrases without the help of lip reading at 1 year of CI use. In summary, the expected perceptual results were the achievement of perceptual category 2 at 3 months from the CI activation, perceptual category 4 at 6 months, and perceptual category 6 at 12 months. The follow-up initially should be very tightly controlled and should be performed in the first year after surgery, at 3, 6, 9, and 12 months and then yearly. The evaluation of perceptual skills and communication has been made by the administration of different tests according to the stage of language development of the child (preverbal stage, transitional stage, and functional stage).

## 3. Results

Between January 1, 1996, and April 1, 2012, at the ENT-Audiology Department of the University Hospital of Ferrara, 620 cochlear implantations were performed. There were 426 implanted children at the time of the present study (who were <18 years).

Reviewing the neuroradiological findings of the 426 implanted children revealed no abnormalities in 283 cases and ear and/or brain anomalies in 143 cases (33.6% of 426). Among these 143 patients (64 females and 79 males), 123 children had unilateral cochlear implantation (68 in the right ear; 55 in the left ear), and 20 underwent bilateral cochlear implantations (3 simultaneously, 17 sequentially). The age of the main study group (143 implanted children) ranged from 9 months and 16 years (mean = 4.4; median = 3.0). These patients showed an average period of cochlear implant use of 74 months.

The CT and MRI scans of 143 children included in the present study were reevaluated, and the following abnormalities were detected: in 69 cases (48.2% of 143), ear malformations were present, of which 55 had bilateral ear involvement; therefore, the implanted ear was necessarily the malformed ear; in 11 children, the malformed ear was the right ear (of which only in one case the malformed side was the implanted side), and in 3 cases, the malformation was detected in the left side (also in this series only one child underwent cochlear implantation in the malformed ear); 74 cases (51.7% of 143) presented only brain anomalies. A total of 45 patients (31.5% of 143) presented either ear or brain abnormalities.  Table 1 shows different aetiologies of hearing loss that we found in our series. Demographic groups and audiological features are resumed in Table 2.

Details of the identified cochleovestibular (inner ear) malformations are presented in Tables 3 and 4.

Finally, Table 5 reports in detail the brain anomalies that were found, with the total number of cases for each type. For statistical purposes, the main group was divided into subgroups, as follows (Table 6).

### 3.1. Postoperative Speech Perception Outcomes

After 3 months of using the cochlear implant, more than half of the patients in the main study group did not achieve the 3th category of perception at the Geers and Moog scale; in the same group, the 50% of the children did not reach the 4th category at the 6-month follow-up; nevertheless, they achieved the 5th category 6 months after (1-year follow-up). Only 2 years after cochlear implant activation, the majority of the patients attain a 6th perceptual category at the Geers and Moog scale (Table 7).

**Figure 6:**
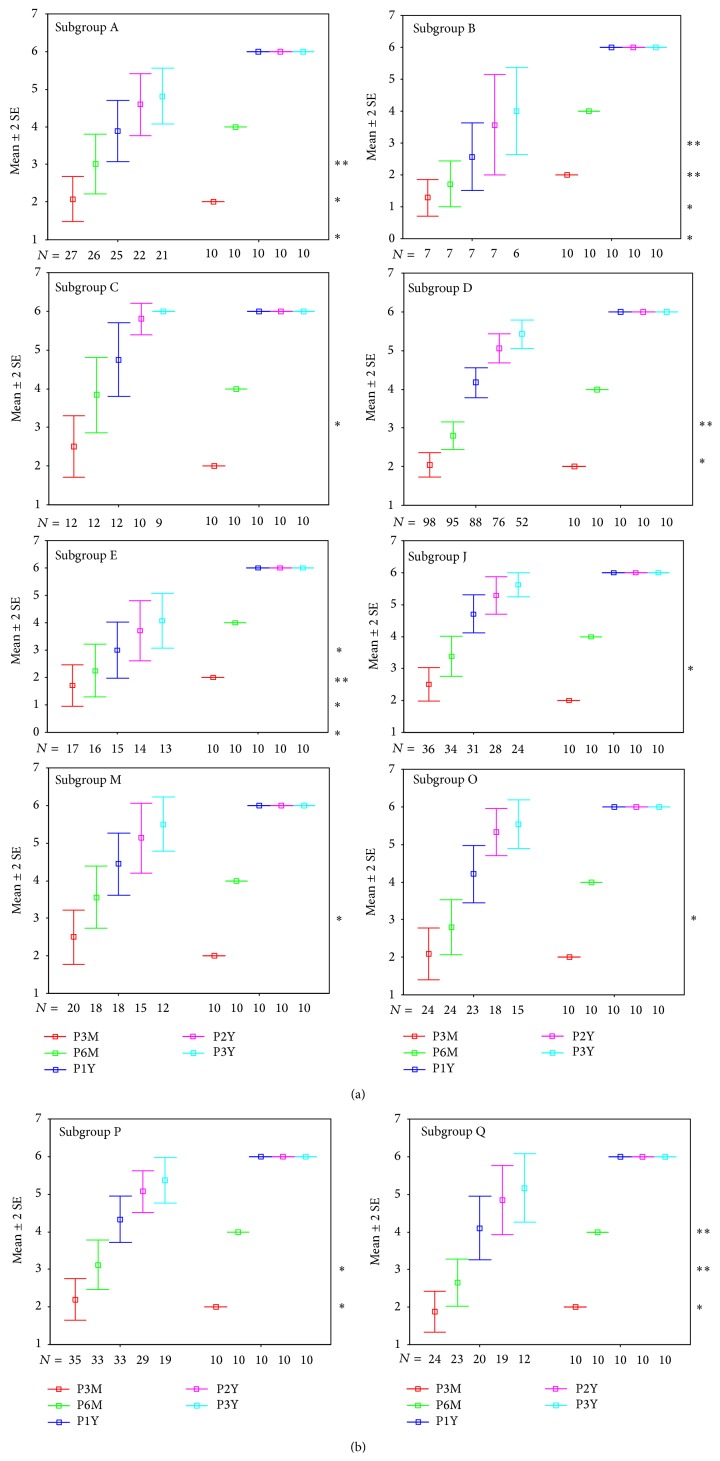
In the figure are reported all of the statistical graphs that were obtained from the comparison between the subgroups A, B, C, D, E, J, M, O, P, and Q and the control group. Colour code for identification (red for the 3-month control, green for the 6-month control, blue for the 12-month control, violet for the 24-month control, and azure for the 36-month control). ^*^ *P* ≤ 0.05; ^**^ *P* ≤ 0.01.

Statistical results are reported in full as the following graphs (Figure 6). These graphs show the comparison between the control group and each of the subgroups; nonetheless, different subgroups were compared.

Graphs are structured as follows: on the abscissa axis are reported the number of cases, and they are distributed over the time of the 3-, 6-, 12-, 24-, and 36-month follow-ups, using a colour code for identification (red for the 3-month control, green for the 6-month control, blue for the 12-month control, violet for the 24-month control, and azure for the 36-month control).

There was a statistically significant difference (*P* ≤ 0.01) between the control group and the subgroup B (patients with internal auditory canal stenosis) at the 6-month follow-up. Similar results were obtained comparing control group and subgroup Q (patients affected by leukomalacia).

At the 1-year and long-term (2-3 years) follow-ups, statistically significant differences (*P* ≤ 0.05) were also found comparing control group and subgroups A, B, D, and E, respectively.

Comparing subgroups C and P we found that the first one achieved the “identification of verbal sounds” 6 months after device activation and the “vowel recognition” in closed set tests 6 months later, while the subgroup P more slowly reached the same perceptual skills. Nevertheless, at the long-term follow-up (2 years later) the results achieved by the two groups are optimal and similar (Table 8).

### 3.2. Outcomes in Patients with Genetic Mutations

**Figure 7:**
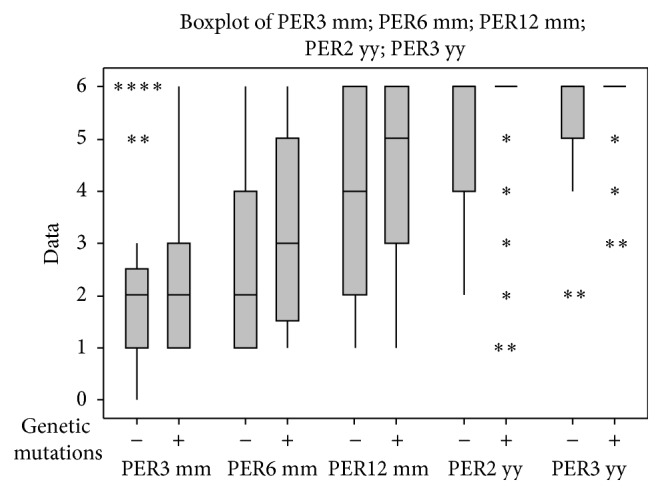
Perceptual outcomes at the 3-month follow-up on the children who underwent genetic investigation; differences between children with genetic mutations (+ = with pathogenic mutations) and children without mutations (− = without pathogenic mutations) are shown in the graphs.

Figure 7 shows the statistical results obtained from patients with or without genetic mutations.

As reported in Table 9, the most common mutation was the 35delG in the* GJB2* gene. All patients with* SLC26A4* mutations presented bilateral EVA. Note that they had mutations on both alleles. Among these mutations, to our knowledge, 2 have never been described before (Q235R e G557D) and 1 was recently reported in one of our scientific publications entitled “Novel Mutations in the* SLC26A4* Gene” [55].

After 3 months of using the cochlear implant, more than half of the patients belonging to the subgroup M (patients who presented EVA) did not achieve the 3th category of perception at the Geers and Moog scale; in the same group, the 50% of the children did not reach the 4th category at the 6-month follow-up; nevertheless, they achieved the 5th category 6 months after (1-year follow-up). Only 2 years after cochlear implant activation, the majority of these patients attained a 6th perceptual category at the Geers and Moog scale (Table 10). There was a statistically significant difference (*P* ≤ 0.05) between the control group and the subgroup M (patients who presented EVA) at the 1-year follow-up (Figure 6).

### 3.3. Outcomes Based on Age at the Time of Surgery

**Figure 8:**
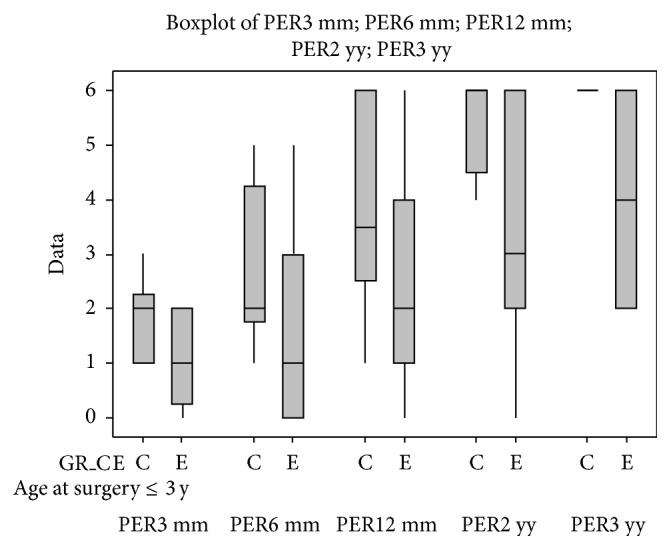
Comparison between the perceptual outcomes at the 3-year follow-up of children who were younger than 3 years old at the time of surgery and who belong to subgroups C and E.

**Figure 9:**
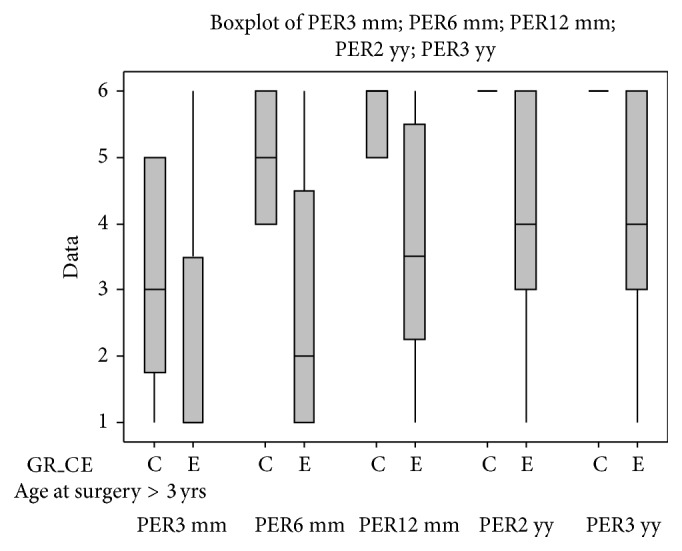
Comparison between perceptual outcomes at the 3-year follow-up of children who were older than 3 years at the time of surgery and who belong to the subgroups C and E.

**Figure 10:**
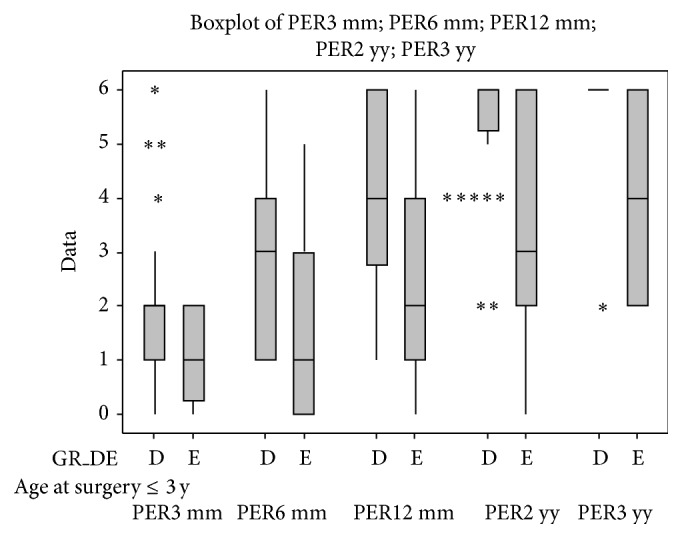
Comparison between perceptual outcomes at the 3-year follow-up of children who are younger than 3 years of age at the time of surgery and who belong to the subgroups D and E.

**Figure 11:**
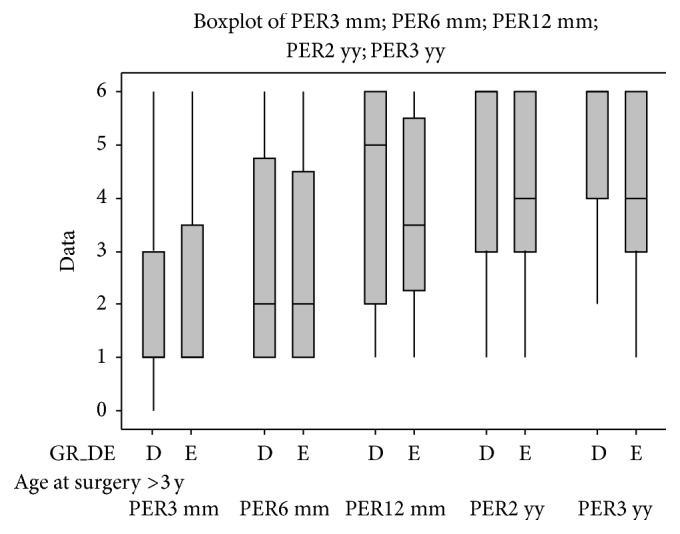
Comparison between perceptual outcomes at the 3-year follow-up of children who are older than 3 years of age at the time of surgery and who belong to the subgroups D and E.

Given the great importance of timing of surgery for the CI outcomes we compared the results obtained from patients with only malformations of the inner ear (subgroup C) and patients with inner ear and concomitant brain abnormalities (subgroup E); then we divided these patients in those who underwent CI within 3 years of age and those who underwent CI after 3 years of age (Figures 8 and 9). Similarly, we compared the patients with only brain abnormalities (subgroup D) and patients with inner ear malformations and concomitant brain abnormalities (Figures 10 and 11).

In our study, it should be noted that the performance after bilateral CI (Table 11) can be influenced down by the fact that results were collected starting from the first CI (dragging effect of the second device over the first one) and in case of a delay in the second CI (sequential surgery), the perceptual skills, at the 1-year follow-up, can still be related to the “effect” of the first CI and likely due to differences in time of using the second device. Comparing unilateral CI and bilateral CI (sequential in almost all cases), it was noted that, at the 1-year follow-up, 1 device allowed vowel recognition in closed set tests in 50% of patients (4th perceptual category), while 2 devices enabled 50% of patients to achieve speech recognition in open set tests (6th perceptual category).

More evident was the difference comparing 1 and 2 CI among patients belonging to the subgroup L in terms of rapidity in achieving the higher perceptual categories; in fact all bilateral cases had reached the “open set recognition” at the 1-year follow-up. In the subgroup L, there were 3 patients with postverbal, simultaneous, and bilateral CI (Table 11).

## 4. Discussion

In the present era, when cochlear implantation is a widely accepted therapy for sensorineural hearing loss, the selection of the patients is still a complex issue demanding close collaboration of experts in all different fields. There is no doubt that thorough radiological evaluation is of enormous importance in malformed inner ears. An exact description and, if possible, classification of the abnormality builds the firm base of further evaluation. The results obtained allow us to affirm that abnormalities of the ear and brain are frequent findings among the cochlear implant candidates; for this reason, the neuroimaging has a fundamental diagnostic role. HRCT scans of the temporal bone help to define the surgical anatomy and provide information about cochlear abnormalities that can aid the surgeon in surgical planning and patient counselling. An absolute contraindication to cochlear implantation detectable by HRCT is the absence of the cochlea in Michel's aplasia. Although HRCT is the gold standard for evaluating most aspects of temporal bone anatomy, MRI is ideal in imaging soft tissue structures such as the membranous labyrinth and nerves. One disadvantage to using MRI in children, though, is the need for sedation [56].

Patients with severe inner ear malformations are expected to perform more poorly than patients with normal cochlea because of the likelihood of fewer spiral ganglion cells and the more complex surgery in malformed ears. Nevertheless, different types of electrode arrays have been introduced to improve the placement of device and to develop speech performance. Because the electrodes may not be confined by scalar anatomy, electrode migration may occur, and individuals with cochlear malformations may require frequent reprogramming of the electrodes. Electrodes that are not intracochlear or that elicit facial nerve stimulation can be eliminated from the “map” as can electrodes that elicit facial nerve stimulation in implanted normal cochleae.

In previously published papers, several authors have shown that the benefits of implants in malformed inner ears are comparable to those gained by deaf children with morphological normal ears [36–38, 57, 58]. Indeed, assert that cochlear implantation can be successfully performed in children with inner ear malformations. The various types of inner ear malformations may have quite different prognoses for good auditory performance. In cases of cochlear ossification, the functional effects remain especially controversial. Predictors of good performance include the constellation of incomplete partition of cochlea: enlarged vestibular aqueduct (EVA), dilated vestibule (i.e., Mondini's malformation), isolated EVA, and partial semicircular canal aplasia. These patients achieve a different level of open set recognition in over the 80% of cases. Children with other cochlear dysmorphologies such as the common cavity or with associated pathologies like the CHARGE association and psychomotor retardation-developmental delay can have poor performance after implantation. Obtaining knowledge of cochlear malformation is especially important in counselling parents before implantation.

Kim et al. [9] observed that cochlear nerve hypoplasia was responsible for poor CI outcome that reduced the chance of an acceptable development of perceptual and linguistic capabilities. Other malformations can be responsible for delay in reaching the higher categories at the Geers and Moog scale. Nevertheless, they found no significant differences between the study group (with inner ear malformations) and control group (without inner ear malformations) at the 2-year follow-up. Loundon et al. [38] reported similar results that are summarized as follows: (1) at the 12-month follow-up, 83% of children achieved 75% of speech recognition in closed-set tests (corresponding to the 5th category at the Geers and Moog scale), (2) only 16% of those patients had obtained the same results during preoperative tests, (3) at the 2-year follow-up, they improved the perceptual abilities, and (4) 64% of children achieved 50% of speech recognition in open-set tests (corresponding to the 6th category at the Geers and Moog scale).

Eisenman and colleagues [57] found that all the subjects of their study showed improved performance on all measures of speech perception over time. Overall, the two groups showed no statistically significant differences in performance at 6 and 24 months. However, subjects with malformed cochleae evidenced slower rates of improvement than did their matched control subjects. Subjects with more severe malformations demonstrated poorer performance, but this may have been attributable to preoperative factors rather than to implant performance [57].

Incesulu and colleagues say that except cochlear or cochleovestibular nerve agenesis, inner ear malformations cannot be accepted as a contraindication for cochlear implantation. Although there can be difficulties during the surgery or in the postoperative period, patients with inner ear malformations can also benefit from cochlear implantation. It is essential that all possible complications and postoperative performance should be discussed with the parents [58].

Although there is controversial data in the literature on the prognostic value of specific factors, such as cochlear malformations and brain abnormalities, we can conclude that these factors do not necessarily affect the outcome of the cochlear implant; in fact, most of the factors that are typically encountered achieve satisfactory results. With the exception of a few special cases, such as stenosis of the internal auditory canal (<2 mm) and the common cavity, or instead the lack of the modiolus, which prevents an optimal pairing between electrodes and cochlear nerve fibres, the results, especially over a long-term period, are comparable to patients without neuroimaging findings, in terms of the achievement of perceptual abilities.

However, we must stress that, especially in the short term period (12 months), the presence of cochlear malformations could slow the attainment of more complete perceptual abilities; even more evident is the effect in the presence of disorders of the central nervous system. Note that the simultaneous presence of the inner ear malformations and anomalies of the brain determines a negative synergistic effect, with the achievement of lower perceptual categories (according to the Geers and Moog score) for the same period of use of the cochlear implant.

In the present study, 28% of implanted children were affected by brain anomalies identified by preoperative neuroimaging. Our results compare well with similar studies. Trimble et al. found central MRI abnormalities in 40% of the patients in their group compared to 20% in the study performed by Lapointe et al. [30]. Lapointe emphasized the importance of neuronal migrational delays resulting in the neurodevelopmental delay and potentially poor outcome from cochlear implantation. In addition to the brain MRI findings mentioned helping to predict speech perception and language outcome, Trimble et al. also commented on the importance of some findings to the anaesthetist (ventriculomegaly, hydrocephalus, Chiari malformation, and intracerebral haemorrhage) [29]. Of the abnormalities detected, 49% were related to known preexisting conditions. By far the most common abnormality detected in 84 patients was white matter changes (70%) and this was found in 13% of all patients investigated. Frequently the white matter changes were related to previous conditions/insults and included infection, ischaemia, hypoxia, and prematurity.

Apart from diagnosing incidental findings, MRI brain can aid in the diagnosis of hearing loss and has been shown to be important in predicting language and speech perception outcomes in patients with kernicterus and cytomegalovirus (CMV) infection as aetiological factors [31, 32, 59]. White matter changes have been shown to be an important determination of abnormal neurodevelopmental outcome and might help predict future problems (seizures and intellectual impairment) in certain patients [60]. The full role of white matter changes in predicting hearing outcome in cochlear implant patients is still unclear.

A further potential advantage of preoperative brain MRI is that it might identify pathology that can be followed up with CT imaging, which is easily accessible postcochlear implantation. This was the case in three of the patients in this series with a lipoma, a hamartoma, and a pinealoma diagnosed on brain MRI whom required further imaging after cochlear implant. In addition, brain MRI will also provide a baseline for comparison with future MRI scans.

The group of patients with bilateral implantation was very heterogeneous by virtue of the great variability in the time of execution of the second cochlear implant; thus, it was not possible to determine the effect in the subgroups. We have focused the investigation on those patients with meningitis and cytomegalovirus infection because the number of cases available for comparison was higher and more homogeneous. Either subgroups (K and L) show a significant improvement after 1 year after the activation of the second cochlear implant.

In children, the most likely cause of cochlear ossification is meningitis. Twenty per cent of children acquire profound bilateral sensorineural hearing loss prior to the age of 3 years; 90 per cent of these cases are meningitic in origin [61]. Labyrinthitis ossificans results from severe inflammation of the inner ear and can be associated with a variety of pathology (advanced otosclerosis, viral or bacterial labyrinthitis, and autoimmune inner ear disease). Labyrinthitis ossification presents one of the greatest challenges to effective, safe cochlear implantation. Green and colleagues demonstrated that ossification due to meningogenic labyrinthitis extended further into the cochlea than ossification due to other causes. The extra bone growth makes the insertion of the electrode a difficult process [62]. In addition, the stimulation of surviving neural elements may be compromised by the bony obliteration, and histopathological reports have shown an association between the degree of bony occlusion and a decreased number of surviving spiral ganglion cells, particularly in cases of bacterial meningitis [63]. For these reasons, patients with labyrinthitis ossificans were often thought to perform at lower levels than those without ossification. In previously published papers [64, 65], several authors have shown that children with postmeningitic hearing loss and cochlear ossification could attain significant benefit from their implant, although children without ossification were likely to perform better. A key factor for success may be the timing of implantation. Ossification may appear as early as 2 months following meningitis, leaving a small time period during which electrode insertion is optimal. As mentioned previously, however, central nervous system sequelae of meningitis are likely to hold sway in determining outcome [66].

Hearing loss is the most common manifestation of congenital CMV infection making CMV a leading cause of nonhereditary congenital hearing loss [67]. The manifestations of CMV infection cover a broad spectrum ranging from asymptomatic to severe systemic disease resulting in significant morbidity and mortality. 90% of infants with congenital CMV are asymptomatic at birth. Despite being asymptomatic at birth, up to 7% of these children will develop sensorineural hearing loss that can be unilateral or bilateral, fluctuating or progressive, and range from mild to profound [68]. Approximately 10% of infants with congenital CMV are symptomatic at birth, and 40% of these patients will develop sensorineural hearing loss [69]. Given the relatively large number of children potentially affected by CMV-related hearing loss and the wide range of manifestations of congenital CMV infection, it is difficult to predict how a child with symptomatic CMV will perform with a cochlear implant. Congenital CMV infection accounted for a significant proportion of patients with SNHL, with an incidence rate comparable with that of* GJB2*-related SNHL.

Previous studies have shown that brain imaging may be a good predictor of adverse neurodevelopmental outcomes. In a study of children with a diagnosis of SNHL, 80% of the CMV positive children had abnormal brain MRI scans compared with only 33% of CMV negative children [70]. Our study demonstrated that certain imaging findings may correlate with worse outcomes after CI. Interestingly, the location of the abnormalities also seemed to correlate with worse perceptive outcomes. The majority of the abnormalities were found in the temporal lobe and parietal lobe. The parietal lobe processes sensory information and houses our language abilities, and the temporal lobe regulates emotion, hearing, language, and learning, which could explain why language outcomes are poorer in children with abnormalities in these regions.

Children with symptomatic congenital CMV appear to derive benefit from CI albeit at a slower rate. In a study of 13 children with symptomatic congenital CMV, 73% of implanted children achieved closed-set word recognition, and 63% achieved open-set word recognition [31, 32]. Ramirez Inscoe and Nikolopoulos demonstrated mixed results for speech perception and intelligibility with 50% of children with congenital CMV performing more poorly than controls, 31% performing similarly, and 19% performing better than controls. These children did, however, derive auditory benefit from cochlear implant [71].

Although our study has limitations including its retrospective nature and small sample size, it provides data that may further efforts to identify factors which may help predict which children with congenital symptomatic CMV will benefit from CI, albeit at a slower rate than other children. The location of central nervous system abnormalities, including gliosis and calcifications, may play a role in audiometric and perceptive outcomes after CI. Early measurements such as brain imaging findings and internal ear imaging findings may allow for more accurate counselling of families regarding anticipated postimplantation performance in children with symptomatic congenital CMV.

Cochlear implants can have impressive effects on a child's language abilities, yet outcomes remain variable across the paediatric population. Numerous studies have thus attempted to identify predictors determining postimplantation communication. So far, relevant factors are age at onset of deafness, age at implantation, length of implant use, amount of residual hearing, duration of deafness, educational mode and resources, and psychosocial elements [2, 4–10].

A clear factor seems to be the age at implantation: children appear to perform better when implanted at earlier stages [72]. On IT-MAIS testing, Robbins and colleagues found that children implanted under the age of 19 months demonstrated faster progress and higher scores than those implanted between the ages of 2 and 3. As Geers found, though, this age advantage disappears after 2 years, implying a critical period of development within the first 2 years of life. At older ages, then, other factors begin to affect implant performance [50].

Although it is known from the literature that the precocity of diagnosis and treatment is an important prognostic factor in the auditory/habilitation, in our study the difference between the patients that, at the time of surgery, were younger than 3 years of age and those who were older than 3 years was not significant or at least fell short of expectations. More specifically, children who received implants within 3 years of age showed the worst results in the early controls (3 months); we cannot exclude a bias of the study due to the fact that children implanted at an early age, at three months after the implant activation, are not able to express perceptual skills that were included in the classification that was used. Nevertheless, at the 6-month follow-up, they started to show the “overtaking” effect in their perceptual performances. The outcome is not significantly different between the two groups, starting from the 1-year follow-up.

Paediatric audiological services should offer children with sensorineural hearing loss testing for mutations in Connexin proteins because mutations in at least two Connexins have been implicated in nonsyndromic hearing (*GBJ2* and* GBJ6*). As mentioned above, the most frequent mutation is found in Connexin 26 encoded by the* GBJ2* gene (35delG), resulting in DFNB1 [73]. Mutations in Connexin 26 result in sporadic and familial severe/profound prelingual hearing loss [24] and account for about 50% of recessive and 10% to 25% of sporadic nonsyndromic hearing loss in Southern European children. An evaluation from United States has shown that nearly 30% have Connexin 26-related hearing loss with all degrees of hearing loss [73] and thus it can be stated that mutations in Connexin 26 may result in all degrees of hearing loss. Thus, it is recommended that all children under 18 years of age with bilateral, permanent, nonsyndromic sensorineural, or mixed hearing loss, irrespective of the level of impairment, for which there is no other explanation, should be offered testing. The initial testing should check for 35delG and/or the other most frequent mutations in the background population. Unless the first screening identifies mutations on both alleles, testing should go on to screening of the entire coding region and splice sites for mutations. In addition, the presence of* GBJ6*/Cx30 deletions should be sought.

The perception at 3, 6, 12, 24, and 36 months shows no significant differences between subjects with genetic mutations (35delG in almost all cases) and patients without mutations. However, the obtained results show how the concomitant presence of malformations of the inner ear in the group of patients with mutation moves away from the expected outcome in patients with the same mutation but without abnormalities of the inner ear. The percentage of patients with mutations in our study group does not differ from the rates observed among patients without neuroimaging findings. This finding means that we are still far from establishing the true contribution of DNA mutations on the anatomy and development of the ear and brain. Therefore, it can be concluded that the detection of an abnormality of the ear or brain should not prevent the execution of genetic testing for mutations that are known to be those that are not associated with malformations (e.g., mutations in the gene* GJB2*).

As part of the protocol for diagnostic evaluation imaging techniques should be used in order to detect aplasia/hypoplasia and/or malformations such as enlarged vestibular aqueduct (EVA). EVA is often found in subjects with Pendred syndrome that is a recessive genetic hearing disorder. Sensorineural hearing loss may be fluctuant or progressive, ranging from mild to profound. The diagnosis of Pendred syndrome (or DFNB4) in such cases depends on analysis of mutations in the* PDS* gene, where the most frequent mutation is the* SLC26A4* [55]. An enlarged vestibular aqueduct remains the most common malformation of the inner ear, but it does not appear to influence the outcome of the cochlear implantation.

There were no significant differences between the group of children who had congenital profound hearing loss and children with progressive hearing loss, in either the short- or the long-term period. Comparing the perceptive outcomes of subgroups C (with inner ear malformations) and E (inner ear malformations with brain abnormalities) shows a significant difference starting from the 6-month follow-up (*P* < 0.05), which becomes more and more evident over time (at a 2- and 3-year follow-up, *P* < 0.01), in favour of those subjects who have only malformations of the inner ear and who have better performances.

We also compared the outcomes of the subgroups D (brain anomalies) and E (inner ear malformations with brain abnormalities) and, in this case, the differences begin to emerge at 12 months from the cochlear implantation (*P* < 0.05); they become more significant over time (at a 2- and 3-year follow-up, *P* < 0.01), in favour of those that have only brain anomalies that have better performances.

Brain anomalies affect the long-term outcome after cochlear implantation the most, especially among children who were older than 3 years at the time of surgery; comparing those children who belong to subgroups C and E showed an increasingly significant difference starting from 6 months (*P* < 0.05) after the cochlear implant activation. Furthermore, children who were older than 3 years and who belonged to subgroups D and E did not show any significant difference, from the underlying “dominant effect” of the brain abnormalities. This “dominant” effect appears to be less evident among the children who are younger than 3 years at the time of surgery, probably due to the greater neuronal plasticity.

## 5. Conclusions

A cochlear implant is a relatively safe and effective treatment for patients who have inner ear malformations and abnormalities of the brain. The aetiology remains unknown in most cases (18.9%). The cytomegalovirus infections are the main form of acquired deficit. The genetic mutation that is the most common among patients in this study remains the 35delG* GJB2* gene. The EVA is still the most common malformation of the inner ear, but it appears to have no specific effect on the outcome of the cochlear implant.

Gliotic injuries and disorders of the white matter brain abnormalities were more frequent, which in general showed a dominant effect on the outcome that was negative. In particular, difficult is fitting the result and the outcome of patients with stenosis of the internal auditory canal or in the presence of malformations with the cochlear absence of the modiolus.

Neuroimaging has been vital for a correct diagnosis and proper preoperative evaluation of cochlear implant candidates. Furthermore, the obtained data could be useful in defining the most appropriate timing of the follow-up, in specific cases and, if necessary, to develop better rehabilitation strategies in the event that the outcomes differ from the expected outcomes.

The CI outcome depends on many variables that range from the age at the time of the surgery to the communication mode. Nonetheless, before demanding a multidisciplinary approach, the otologist (or audiologist) has the responsibility to verify the correct functioning of the device, requiring, if necessary, a manufacturer's report to rule out technical failure. Afterwards, a clinical team should manage such unsatisfactory performances, after CI, starting from a self-review process of the applied paths (in terms of auditory rehabilitation or speech training), in order to detect errors in their settings. If there are persisting doubts concerning with the CI results after a technical and methodological review for challenging cases, the first functional and aetiological diagnosis should be reconsidered and reevaluated by a multidisciplinary team. Cooperation between parents, school administrators, teachers, and speech specialists is also vital to the success of the child.

Although further studies are necessary for the identification of predictive factors, especially in challenging cases, the results of the present study have confirmed the need to carry out diagnostic, therapeutic, and rehabilitation processes in specialised centres with extensive and proven experience.

## Supplementary Material

Supplemental material is a rich atlas of neuroimaging findings. Specific conditions are comprehensively illustrated with succinct pertinent text. Figures S1 and S2 show normal inner ear structures. The subsequent figures are arranged from the most common to the most rare anomalies of the brain and of the ear that were found in the paediatric population studied.

## Figures and Tables

**Table 1:** Suspected main aetiology of hearing loss among 143 children who underwent cochlear implantation and presented ear or brain anomalies.

Aetiology	Number of patients	%
Unknown	27	18.9
Cytomegalovirus infection	26	18.2
*GJB2* mutations	24	16.8
Acquired conditions (prematurity, perinatal suffering/icterus)	16	11.2
Enlarged vestibular aqueduct (EVA)	14 (4 Pendred syndromes)	9.8
Cochleovestibular malformations	14	9.8
Meningitis/encephalitis	12/1	9.1
CHARGE association	3	2.1
Hydrocephalus	2	1.4
Waardenburg syndrome (with dilated vestibule and bilateral cochlear dysplasia)	1	0.7
Möbius syndrome (with microtia and facial nerve aplasia)	1	0.7
Williams syndrome (with EVA and semicircular canal dysplasia)	1	0.7
Other conditions (neurosurgery)	1	0.7
Total	**143**	**100**

**Table 2:** Demographic, clinical and audiological data and aetiologies of hearing loss.

	External ear malformations (total *N*° = 3 cases)	Middle ear malformations (total *N*° = 18 cases)	Inner ear malformations (total *N*° = 30 cases)	Brain anomalies (total *N*° = 119 cases)	Without brain anomalies (total *N*° = 24 cases)
Sex (female : male)	1 : 2	9 : 9	11 : 19	55 : 64	9 : 15
Mean age (in years) at the time of surgery	3.0	3.0	4.4	4.0	7.0
Mean period (in months) of using cochlear implant	78	73	74	75	71
Implanted ear (°)	2L, 1R	8L, 7R, 3B	11L, 15R, 4B	42L, 61R, 16B	13L, 7R, 4B
% of progressive hearing loss	—	22.2%	30.0%	45.3%	54.1%
Cytomegalovirus infections	—	3	2	26	—
Meningitis	—	—	1	11	1
Genetic mutations	—	2	5	21	3
Syndromes	3	3	5	6	4
Unknown aetiology	—	3	—	26	1
Other conditions (^*^)	—	2	2	19	1

°(R = right; L = left; B = bilateral); ^*^(infant cerebral palsy, prematurity, perinatal suffering, hydrocephalus, ischemia, and neonatal icterus).

**Table 3:** Anatomic distribution of inner ear malformations.

	Cochlear malformations (total *N*° = 21 cases)	Vestibular and semicircular canal malformations (^§^) (total *N*° = 24 cases)	Abnormal internal auditory canal (^•^) (total *N*° = 15 cases)	EVA (*N*° = 21 cases)
Sex (female : male)	6 : 15	10 : 14	10 : 5	10 : 11
Mean age (in years) at the time of surgery	4	4	5	7
Mean period (in months) of using cochlear implant	75	77	84	54
Implanted ear (°)	8L, 11R, 2B	6L, 13R, 5B	7L, 8R	12L, 7R, 2B
Malformed side (°)	1L, 2R, 18B	1L, 2R, 21B	1R, 14B	1L, 3R, 17B
% of progressive hearing loss	28.6%	41.6%	26.6%	71.4%

°(R = right; L = left; B = bilateral); ^§^(6 hypoplasias; 7 dilatations; 3 aplasias); ^•^(7 stenosis; 8 dilatations).

**Table 4:** Types of cochlear malformations.

	Common cavity	Cochlear hypoplasia (total *N*° = 6 cases)	Incomplete partition type 1 (total *N*° = 2 cases)	Incomplete partition type 2 (total *N*° = 7 cases)	Incomplete partition type 3 (total *N*° = 2 cases)	Cochlear basal turn dysplasia (total *N*° = 4 cases)
Sex (female : male)	—	2 : 4	0 : 2	3 : 4	1 : 1	0 : 4
Mean age (in years) at the time of surgery	—	5	3	5	7	2
Mean period (in months) of using cochlear implant	—	59	82	85	77	73
Implanted ear (°)	—	2L, 4R	1L, 1R	2L, 4R, 1B	1L, 1R	2L, 1R, 2B
Malformed side (°)	—	1L, 1R, 4B	2B	1R, 6B	2B	4B
% of progressive hearing loss	—	40%	—	28.6%	50%	25%

°(R = right; L = left; B = bilateral).

**Table 5:** Brain anomalies that were found among 143 implanted children.

Type of lesion/malformation	Total number of cases among 143 implanted children
Gliosis	32
Dysmyelination/demyelination	25
Leukomalacia	24
Pineal cyst	9
Arnold-Chiari malformation (type 1)	7
Cerebellar hypoplasia	6
Cortical dysplasia	5
Calcifications	3
Arachnoid cyst	3
(External) Hydrocephalus	3
Dilated lateral ventricles	2
Corpus callosum hypoplasia	2
Dilated fourth ventricle	2
Trigonocephaly	2
Facial nerve aplasia	1
Pinealoma	1
Hamartoma	1
Hydrocephalus	1
Bulbar atrophy	1
Cisternal dilatation	1
Malignant neoplasm of encephalon (after surgery)	1
Pellucid septum cyst	1
Dilated subarachnoid space	1
Focal ischemic lesions	1
Microcephaly	1
Lipoma	1
(Occipital) Myelomeningocele	1
Leukodystrophy	1
Pachygyria	1
Temporal lobe hypoplasia	1

**Table 6:** Different subgroups were defined to implement the statistical analysis.

Groups	Inclusion criteria	Number of cases
Main study group	Cochlear implant recipients who were less than 18 years of age at the time of surgery and who presented neuroradiological findings at preoperative neuroimaging investigations	**143**
Subgroup A	Patients with inner ear malformations (with or without brain anomalies)	**23**
Subgroup B	Patients with internal auditory canal stenosis	**7**
Subgroup C	Patients with only inner ear malformations (without brain anomalies)	**13**
Subgroup D	Patients with only brain anomalies (without inner ear malformations)	**102**
Subgroup E	Patients with inner ear malformations and brain lesions or abnormalities (with brain anomalies)	**17**
Subgroup F	Monolateral CI	**123**
Subgroup G	Bilateral CI	**20**
Subgroup H	<3 years of age at the time of surgery	**61**
Subgroup I	>3 years of age at the time of surgery	**82**
Subgroup J	Patients with genetic mutations	**35**
Subgroup K	Cytomegalovirus	**26**
Subgroup L	Meningitis (as the cause of the hearing loss)	**13**
Subgroup M	Patients who presented EVA	**21**
Subgroup N	CHARGE association	**3**
Subgroup O	Demyelination	**25**
Subgroup P	Gliosis	**36**
Subgroup Q	Leukomalacia	**25**
Subgroup R	Patient with only cochlear malformations	**9**

**Table 7:** Perceptual outcomes of the main study group with a cochlear implant at the six-year follow-up.

Controls	*N*	Mean	SD	Percentiles		
				(25°	50°	75°)
3 m	133	2,113	1,579	1,00	2,00	3,00
6 m	127	2,945	1,844	1,00	3,00	4,00
1 y	117	4,120	1,890	2,00	5,00	6,00
2 y	100	4,940	1,693	4,00	6,00	6,00
3 y	70	5,243	1,408	4,75	6,00	6,00
4 y	44	5,432	1,301	6,00	6,00	6,00
5 y	29	5,896	0,409	6,00	6,00	6,00
6 y	24	6,000	0,000	6,00	6,00	6,00

**Table 8:** Comparison of the perceptual outcomes at the 3-month follow-up on congenital hearing loss without progression (C) and progressive hearing loss (P).

Controls		*N*	Mean	SD	Percentiles		
					(25°	50°	75°)
3 m	P	63	2,19	1,68	1	1	3
	C	74	2,05	1,47	1	2	2
6 m	P	60	3,13	1,96	1	3	5
	C	72	2,77	1,68	1	2	4
12 m	P	58	4,32	1,98	2	5,5	6
	C	67	3,98	1,79	3	4	6
2 y	P	53	4,96	1,70	4	6	6
	C	56	5,018	1,64	4	6	6
3 y	P	37	5,21	1,51	5	6	6
	C	44	5,40	1,26	6	6	6

**Table 9:** Mutations that were found among 143 implanted children.

Gene	Mutation	*N*°
*GJB2 *	35delG/35delG	15
*GJB2 *	35delG/R184P	4
*GJB2 *	35delG/167delT	1
*GJB2 *	35delG/R143V	1
*GJB2 *	V27I/E114G	1
*GJB2 *	VS1+1G>A/delE120	1
*SLC26A4 *	G209V/Q235R	1
*SLC26A4 *	L445W/G557D	1
*SLC26A4 *	R409H/IVS2+1delG	1
*SLC26A4 *	R409H/Q235R	1
*MT-RNR1 (MIT DNA) *	C722X (homoplasmy)	1

**Table 10:** Perceptual outcomes of subgroup M (EVA) at the 3-year follow-up.

Controls	*N*	Mean	SD	Percentiles		
				(25°	50°	75°)
3 m	20	2,50	1,60	1	2	3
6 m	18	3,55	1,75	2	3	5,25
12 m	18	4,44	1,75	3	5	6
2 y	15	5,13	1,80	6	6	6
3 y	12	5,50	1,24	6	6	6

**Table 11:** Comparison between perceptual outcomes at 1-, 3-, and 5-year follow-ups of children who have 1 or 2 cochlear implants among the main group and subgroups K and L.

	Percentile	One-year follow-up			Three-year follow-up			Five-year follow-up		
		25°	50°	75°	25°	50°	75°	25°	50°	75°
Unilateral CI	Main group (*n*° 123)	2	4.5	6	4.5	6	6	6	6	6
	meningitis (*n*° 10)	1	2	4	2.5	5	6	6	6	6
	Cytomegalovirus (*n*° 21)	3	6	6	6	6	6	6	6	6

Bilateral CI	Main group (*n*° 20)	3	6	6	6	6	6	6	6	6
	meningitis (*n*° 3)	6	6	6	6	6	6	6	6	6
	Cytomegalovirus (*n*° 5)	3	5	6	6	6	6	6	6	6
