# Supplementary material for: Cochlear Implant Outcomes and Genetic Mutations in Children with Ear and Brain Anomalies
Source: Biomed Res Int. 2015 Jul 5;2015:696281. doi: 10.1155/2015/696281 (PMC4506828; doi:10.1155/2015/696281)
Supplement: Supplementary file 1 — Supplemental material is a rich atlas of neuroimaging findings. Specific conditions are comprehensively illustrated with succinct pertinent text. Figures S1 and S2 show normal inner ear structures. The subsequent figures are arranged from the most common to the most rare anomalies of the brain and of the ear that were found in the paediatric population studied. [file 696281.f1.pdf]

NEUROIMAGING ATLAS

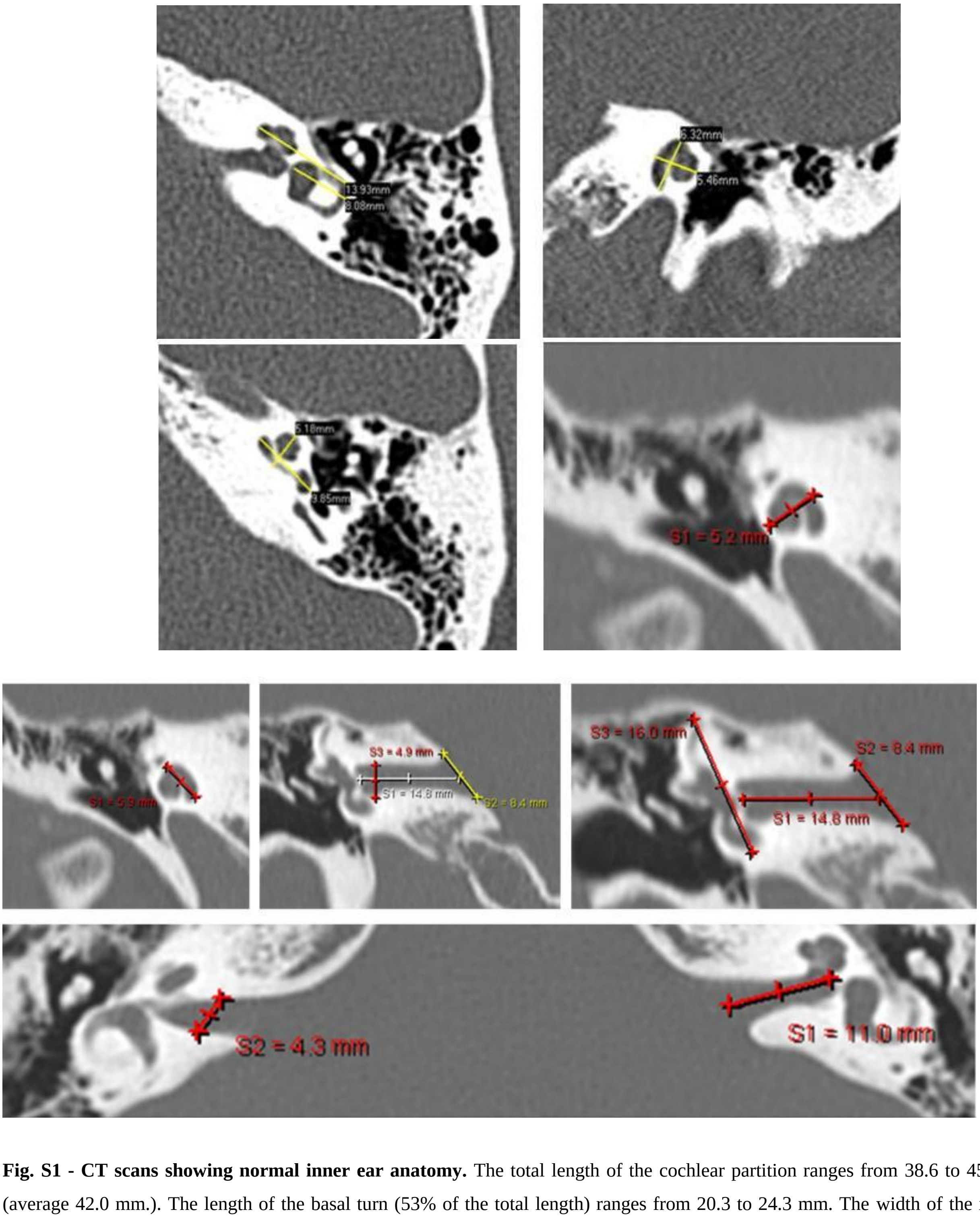

Fig. S1 - CT scans showing normal inner ear anatomy. The total length of the cochlear partition ranges from 38.6 to 45.6 mm (average 42.0 mm.). The length of the basal turn (53% of the total length) ranges from 20.3 to 24.3 mm. The width of the internal auditory canal is between 4.85 and 5.02 mm. The height ranges from 4.39 to 4.62 mm and the length from 11.22 to 11.44 mm [48].

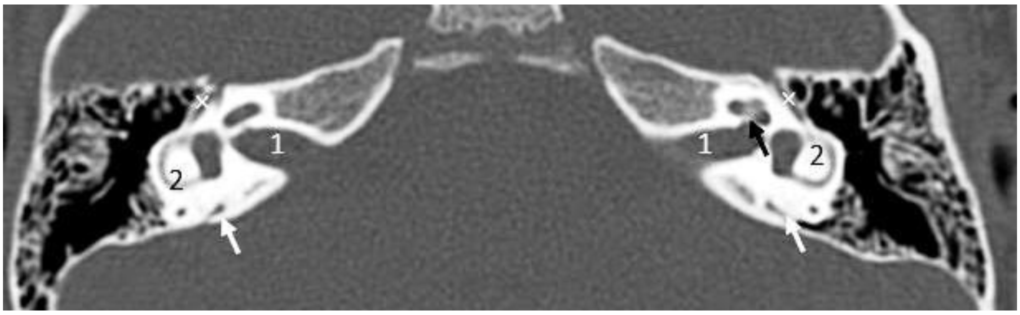

Fig. S2 - Normal ear, CT scan, axial image: 1 = internal auditory canals; 2 = lateral semicircular canals; white arrows = vestibular aqueducts; black arrow = cochlea (modiolus); white X = facial nerves.

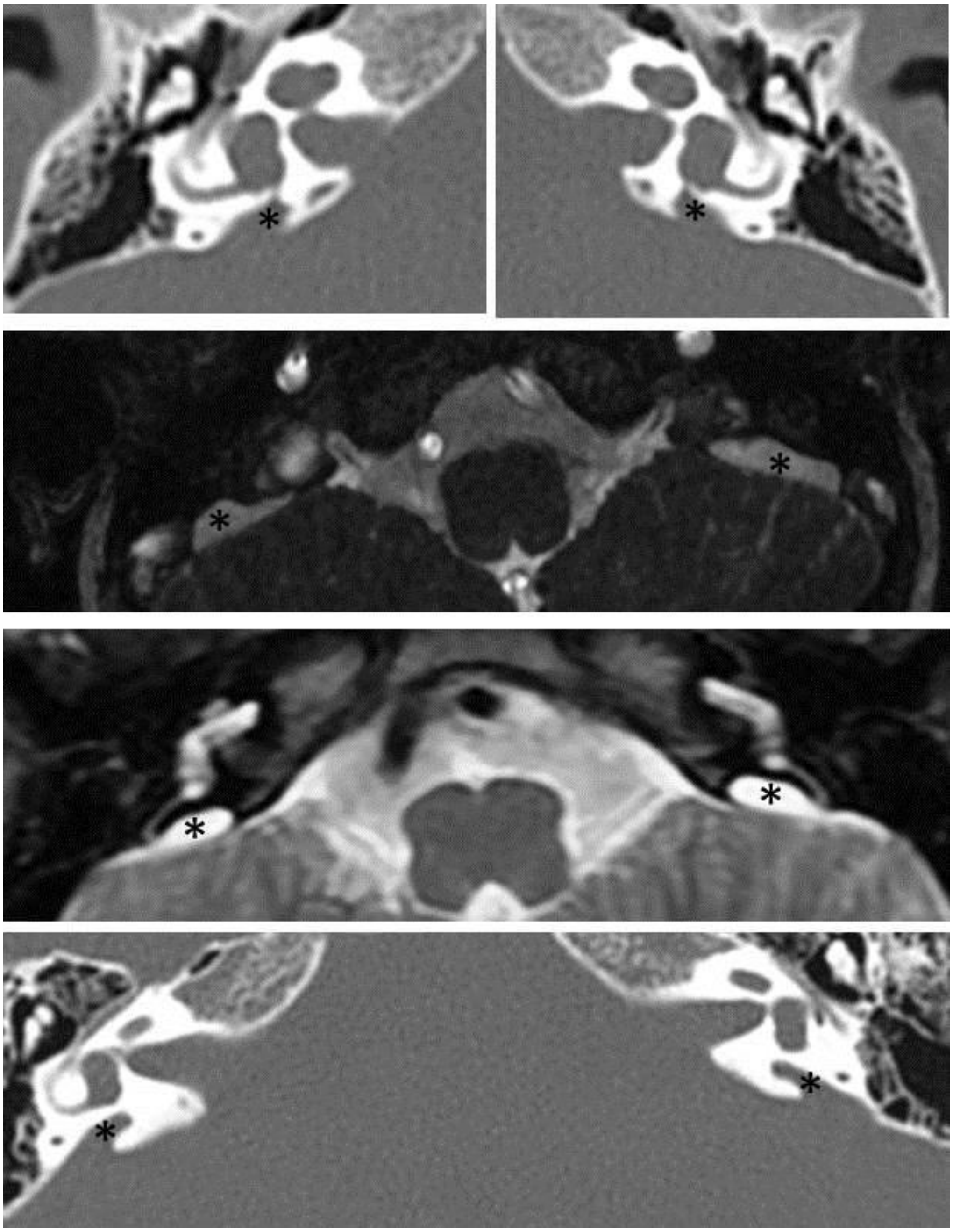

Fig. S3 - CT and MRI temporal bone; black asterisks = enlarged vestibular aqueduct (EVA).

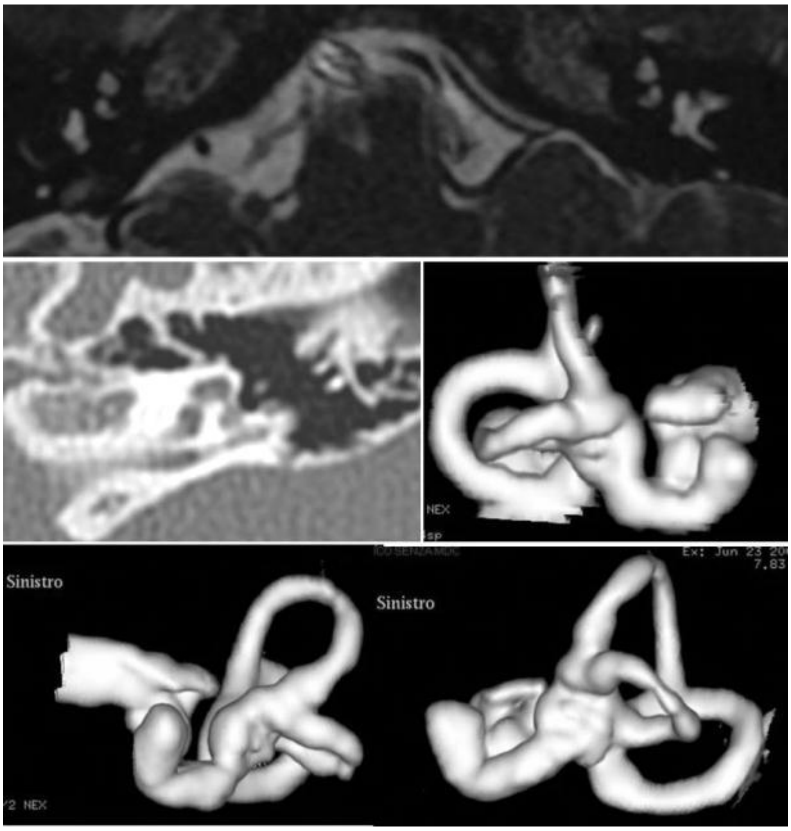

Fig. S4- CT and MRI temporal bone scans with 3-D MR imagines showing bilateral hypoplastic cochlea with normal basal turn.

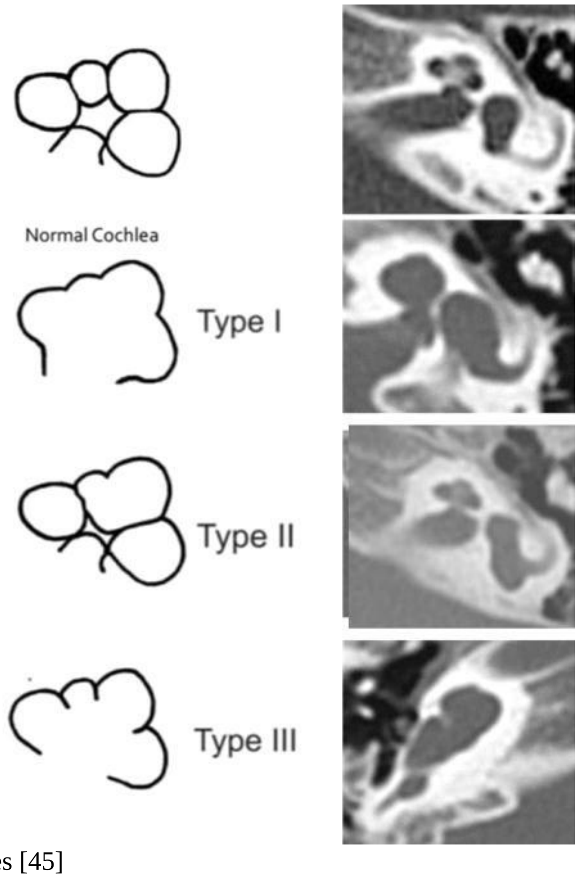

Fig. S5 - Incomplete Partition Types [45]

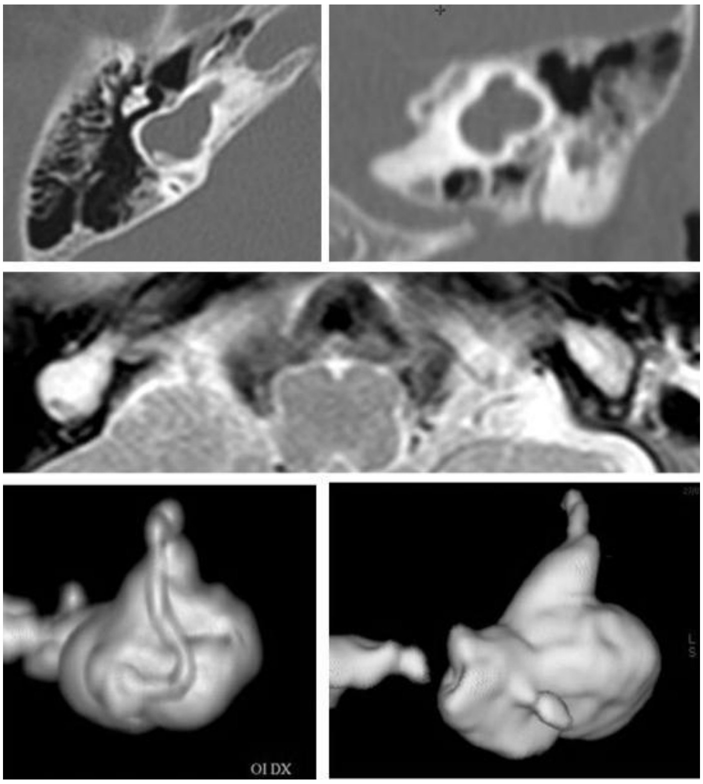

Fig.S6 - CT and MRI temporal bone, 3-D MR imagines. The bony labyrinth presents as one large cystic structure without differentiation of the cochlea or the semicircular canals, there are only small bud like recesses anteriorly and posteriorly shown, common cavity.

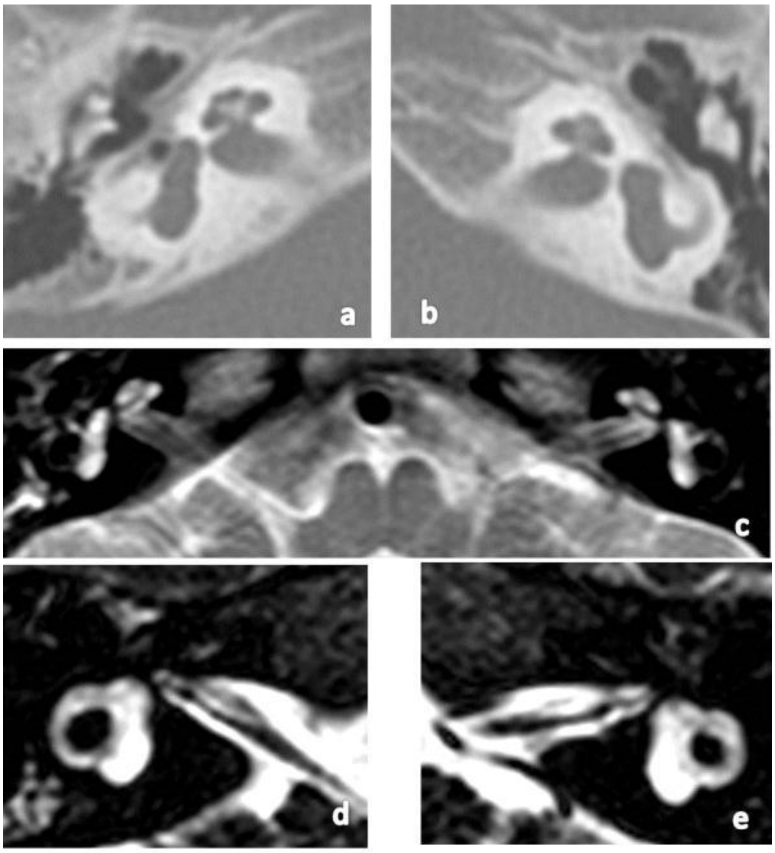

Fig. S7 - CT and MRI temporal bone. Hypoplastic cochleae and dilated saccules**.**

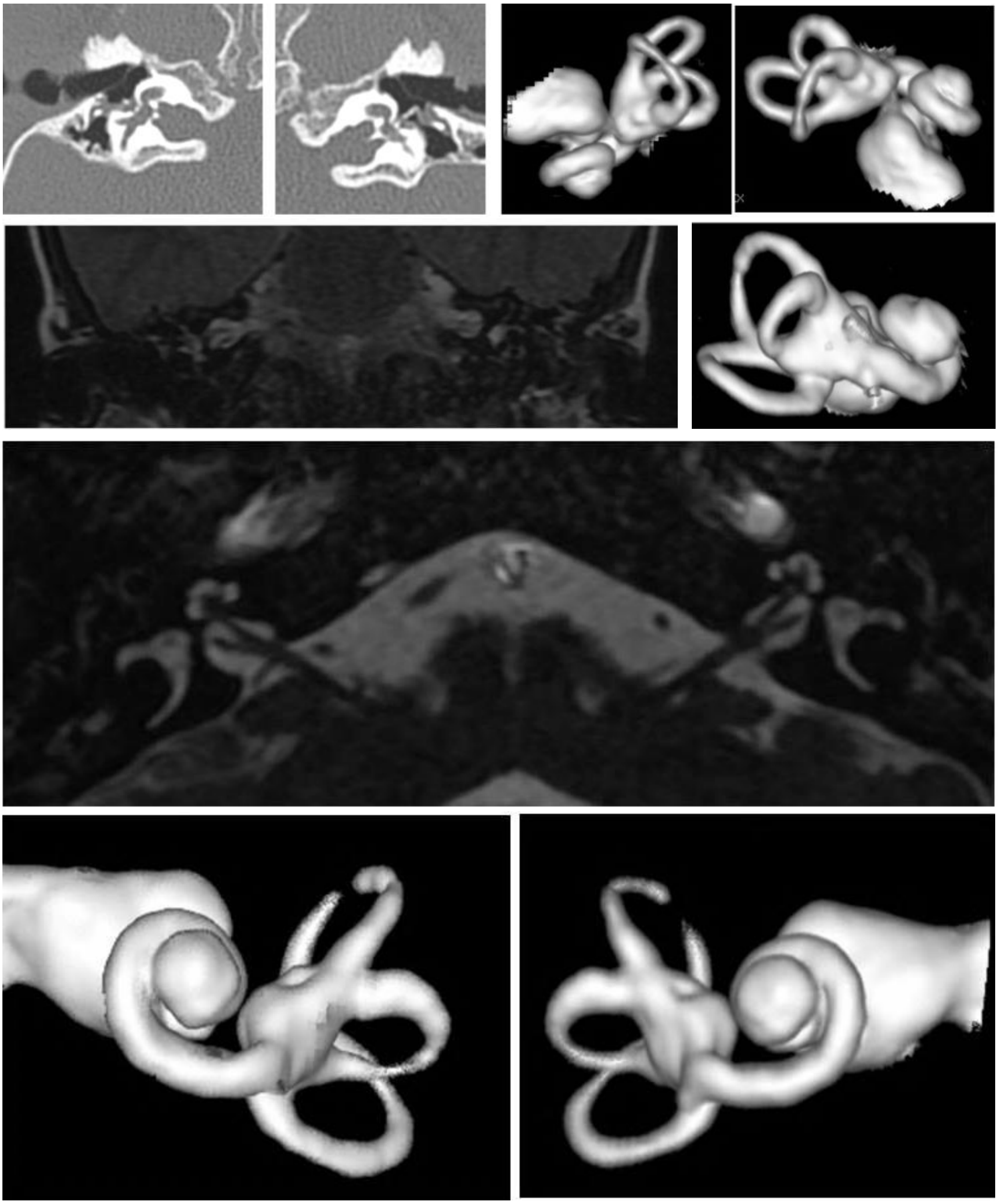

Fig. S8 - CT and MRI temporal bone, 3-D MR imagines; bulbous dilatation of the fundi of the internal auditory canals and congenital stenosis of superior semicircular canals.

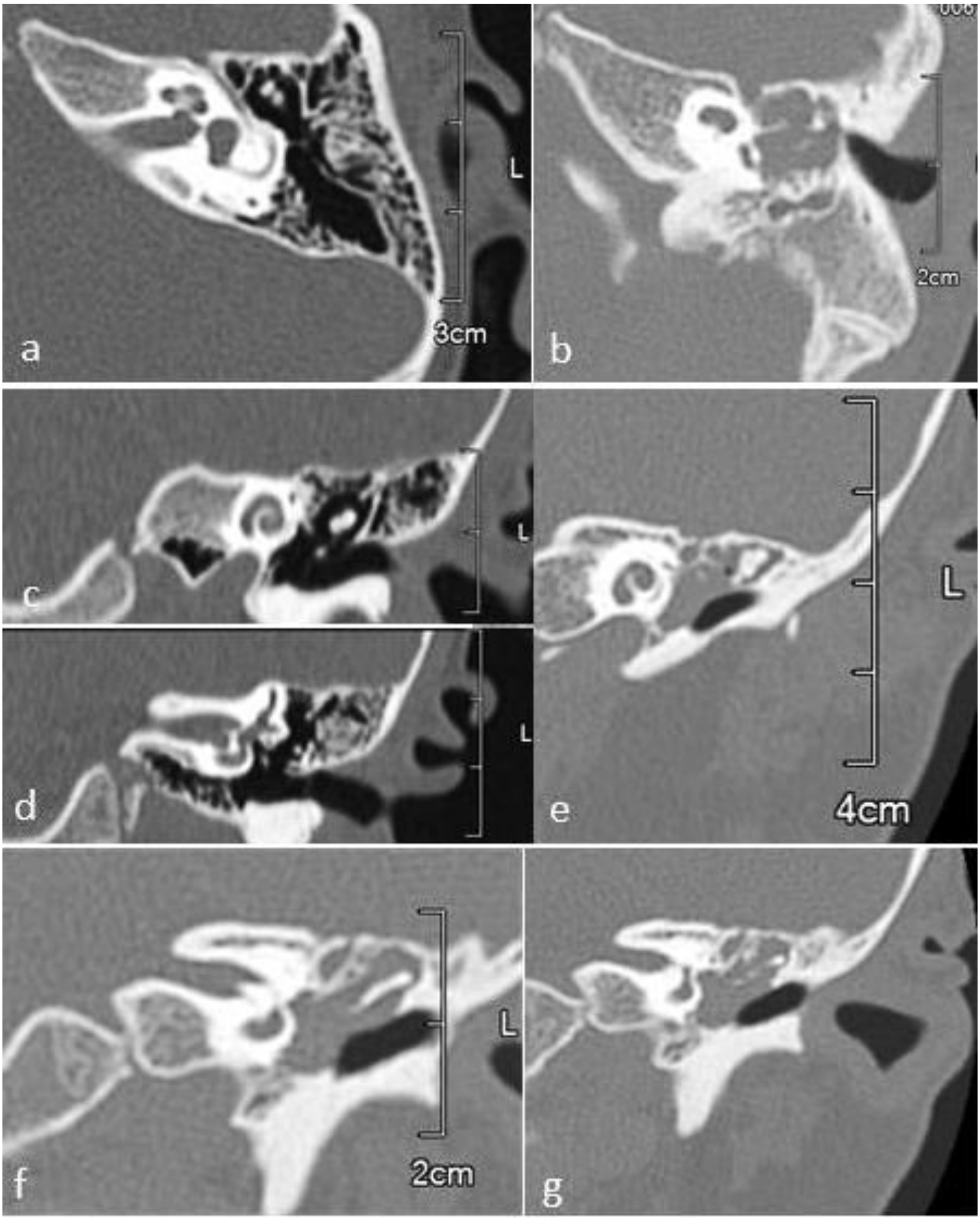

Fig. S9 - Temporal bone CT scan - Axial and coronal images: a), c), d), normal ear; b) hypoplastic/cystic cochlea, modiolus absent, anomalous facial nerve course, absence of the vestibular system; c) malformed and dislocated ossicular chain, cochlear malformation (hypoplasia); f) g) absent semicircular canals.

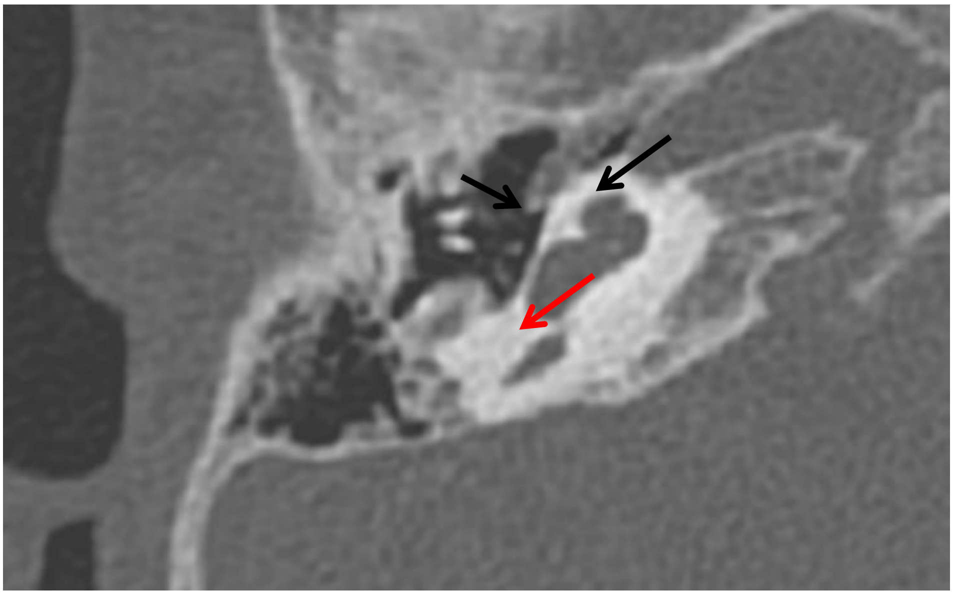

Fig. S10 - Axial CT scan showing an incomplete partition type III (black arrows) and cystic aspect of the vestibular system (red arrow).

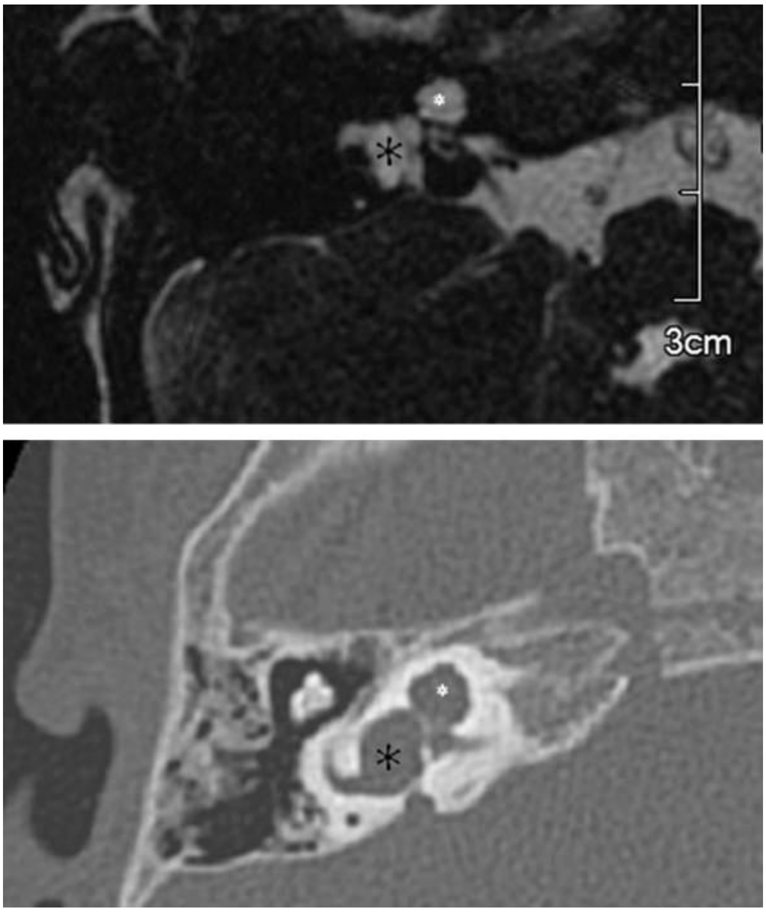

Fig. S11 - MRI and CT scans of temporal bone (right ear) revealing a cystic cochlea (white asterisk) and dilated vestibule (black asterisk).

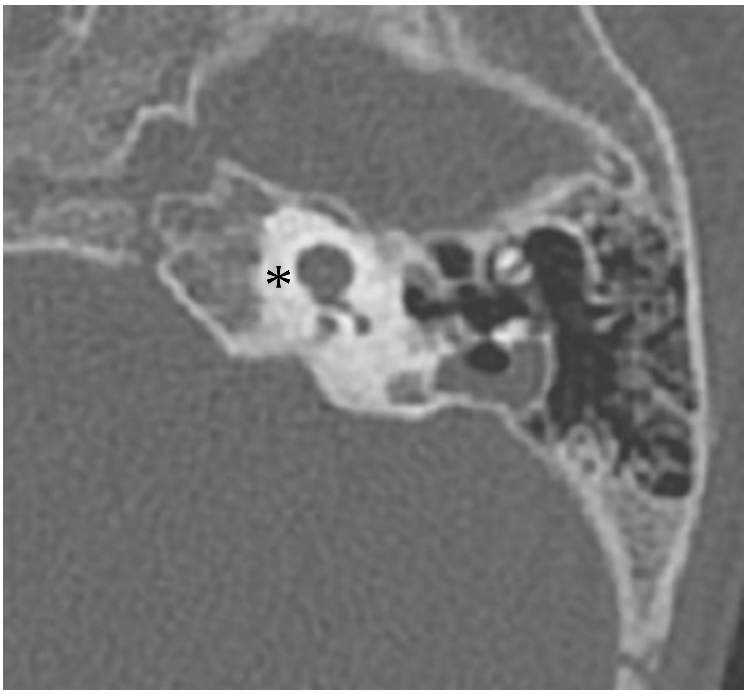

Fig. S12 - CT scan, axial image of temporal bone: incomplete partition type I.

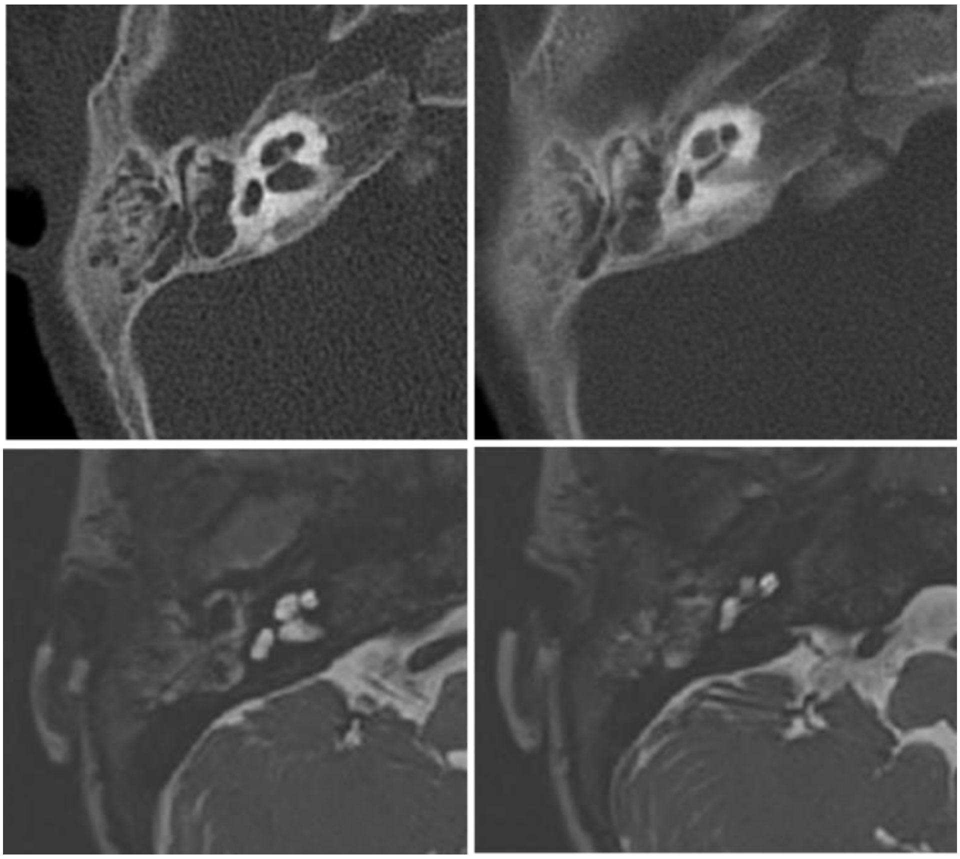

Fig. S13 - CT and MRI revealing an incomplete partition type II in a patient affected by CHARGE association.

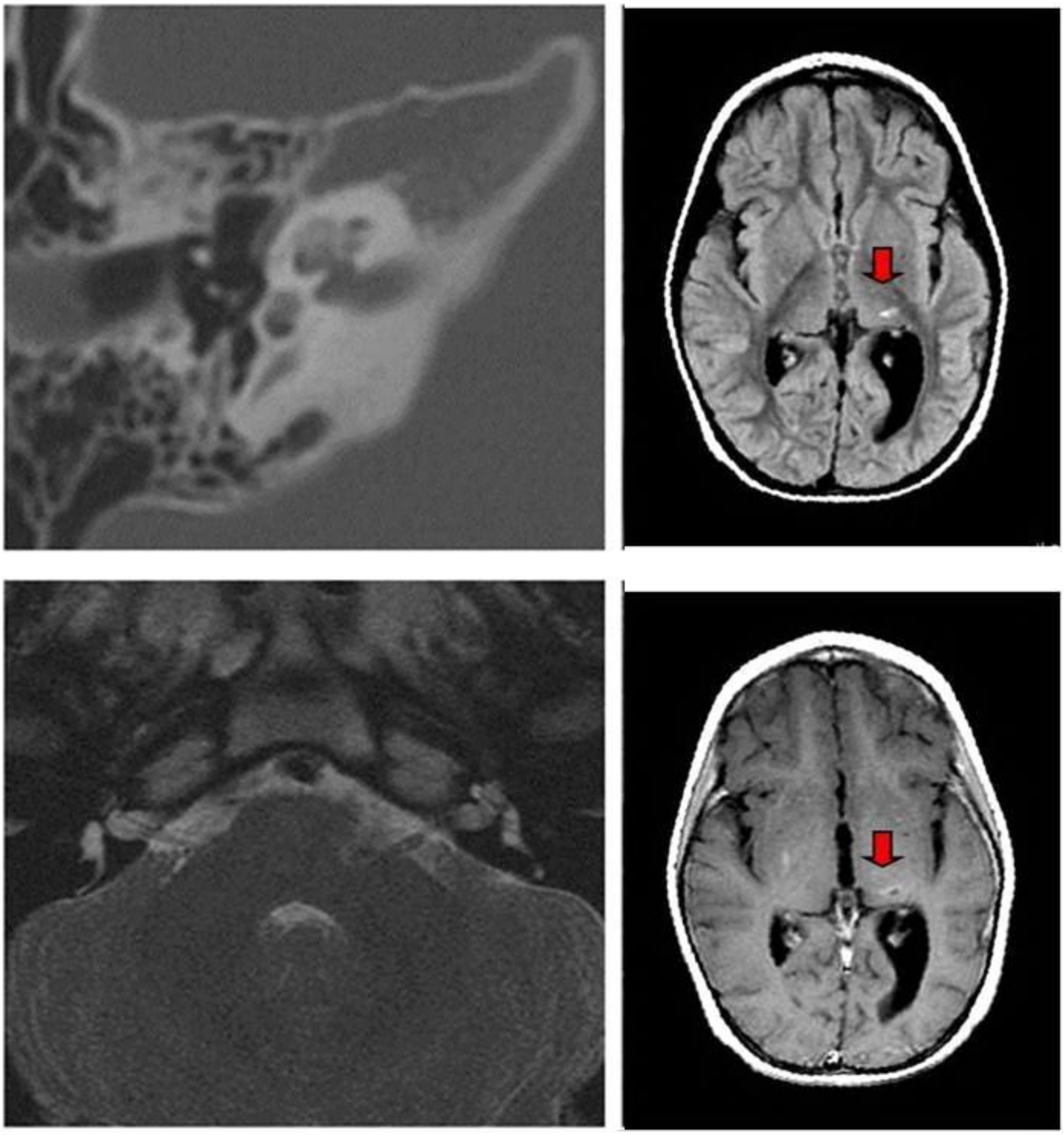

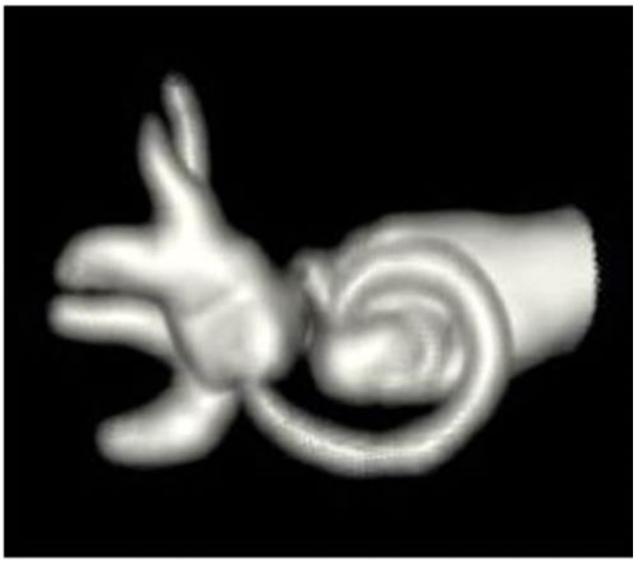

Fig. S14 - CT and MRI scans. After-effects of CMV meningo-encephalitis, with patchy lesions of the white matter (red arrow) and dilation of the left lateral ventricle. 3-D MR image of a case of semicircular canal occlusion, complication of CMV meningo-encephalitis

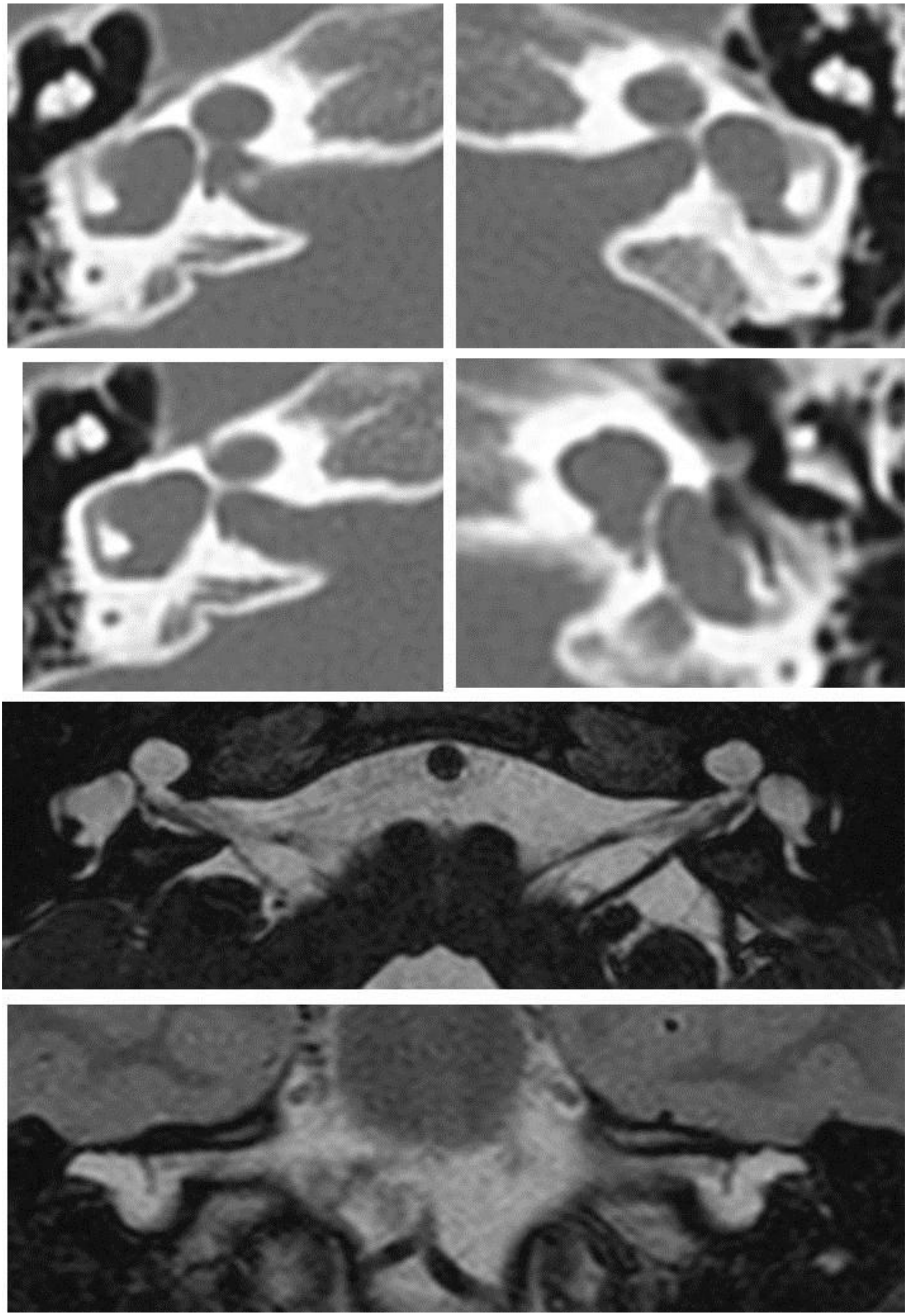

Fig. S15. – Cystic cochleae with apical turn aplasia, dilated vestibules, hypoplastic semicircular canals.

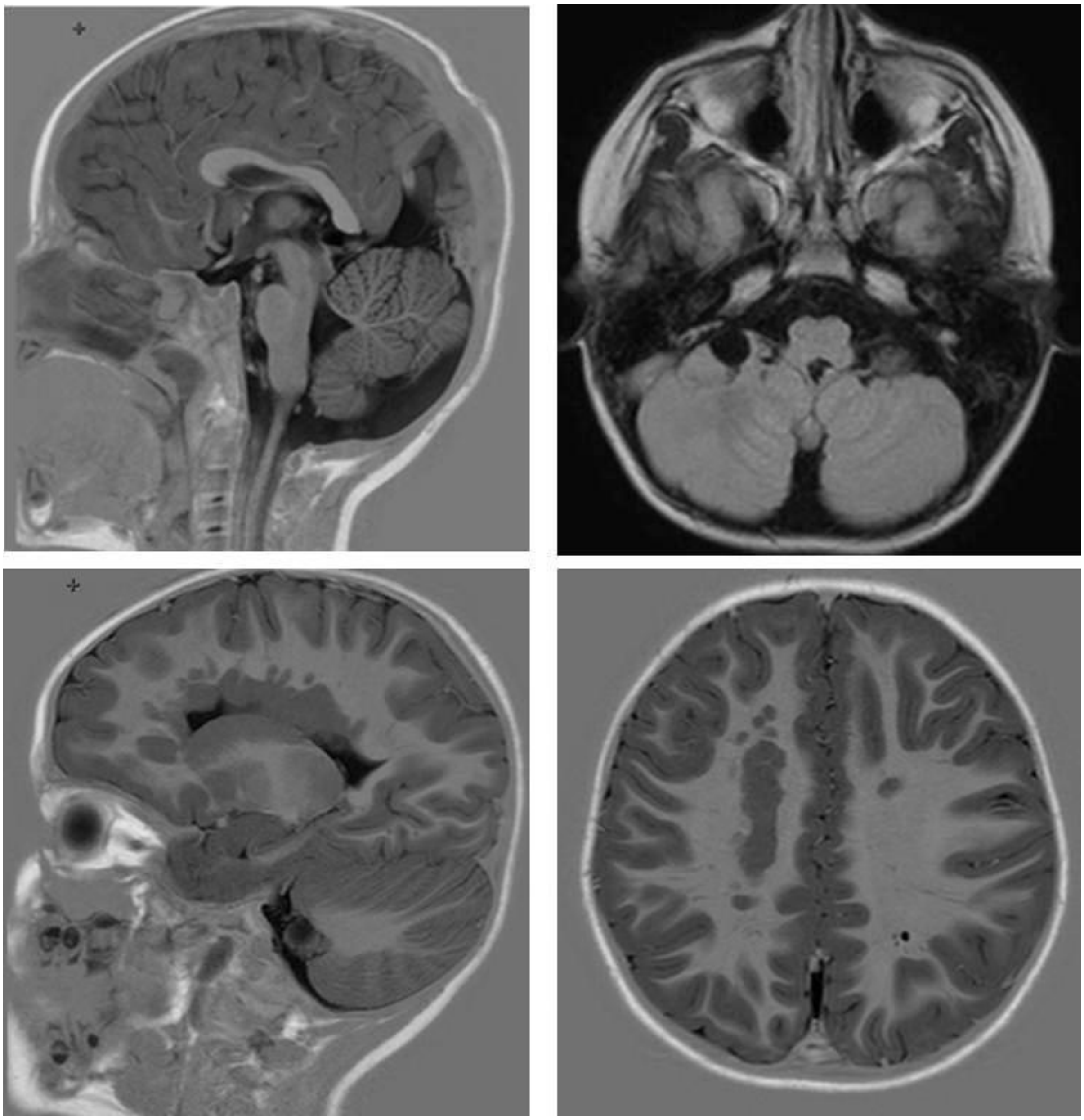

Fig. S16 – MRI; dilated cisterna magna (or cerebellomedullary cistern); hypoplasia of the corpus callosum; dilated fourth ventricle; gray matter heterotopia; post-surgical anatomy of occipital myelomeningocele closure.

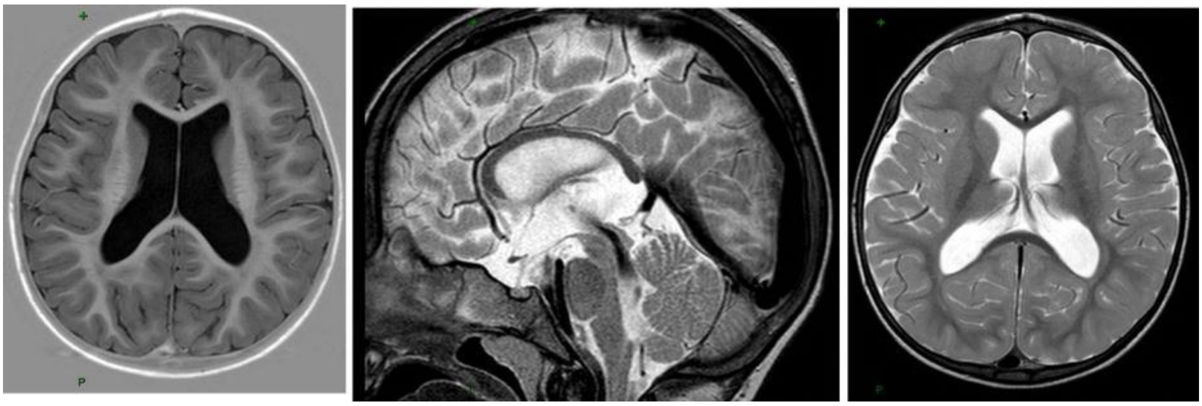

Fig. S17 MRI scans. hypoplasia of the corpus callosum; dilated lateral ventricles (external hydrocephalus

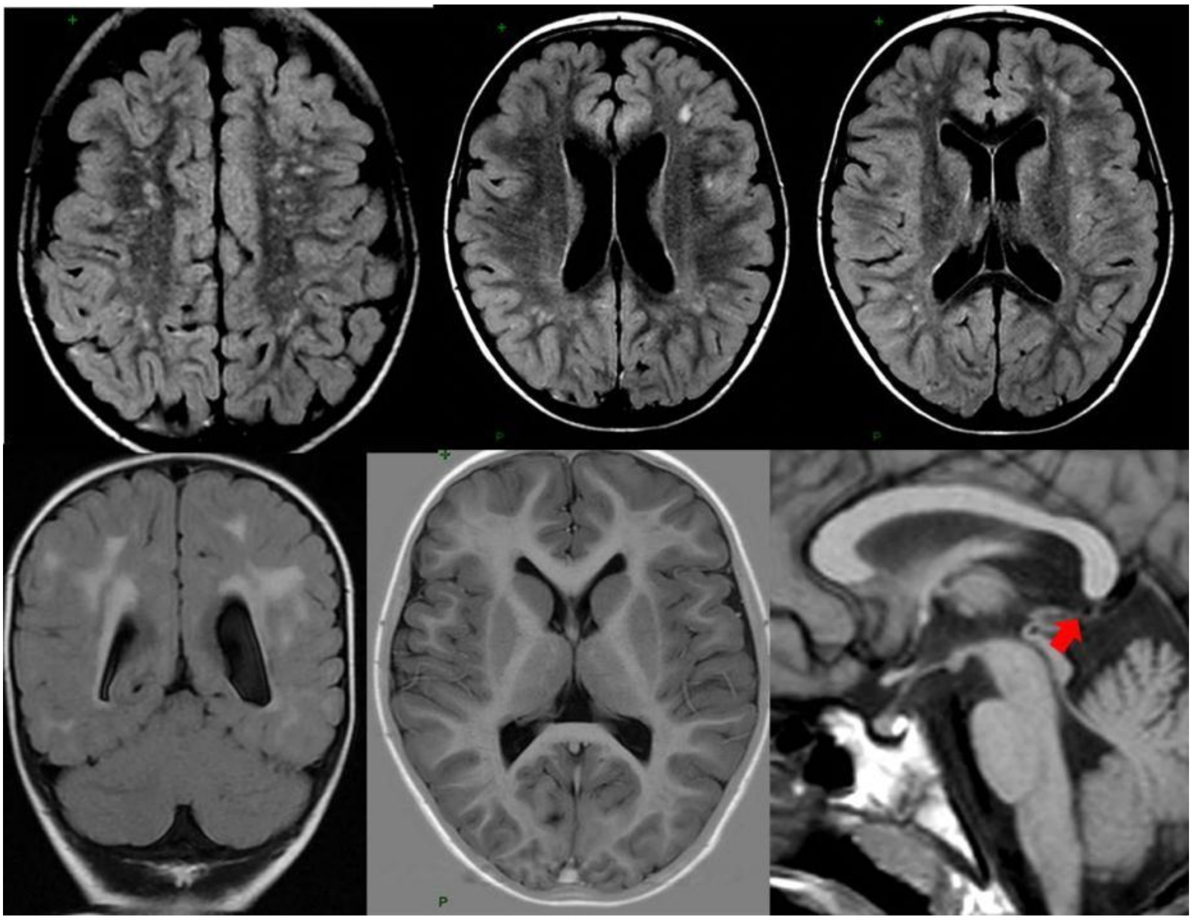

Fig. S18 MRI scans. CMV meningo-encephalitis, with patchy lesions of the white matter and dilation of the lateral ventricle; red arrow: pineal cyst.

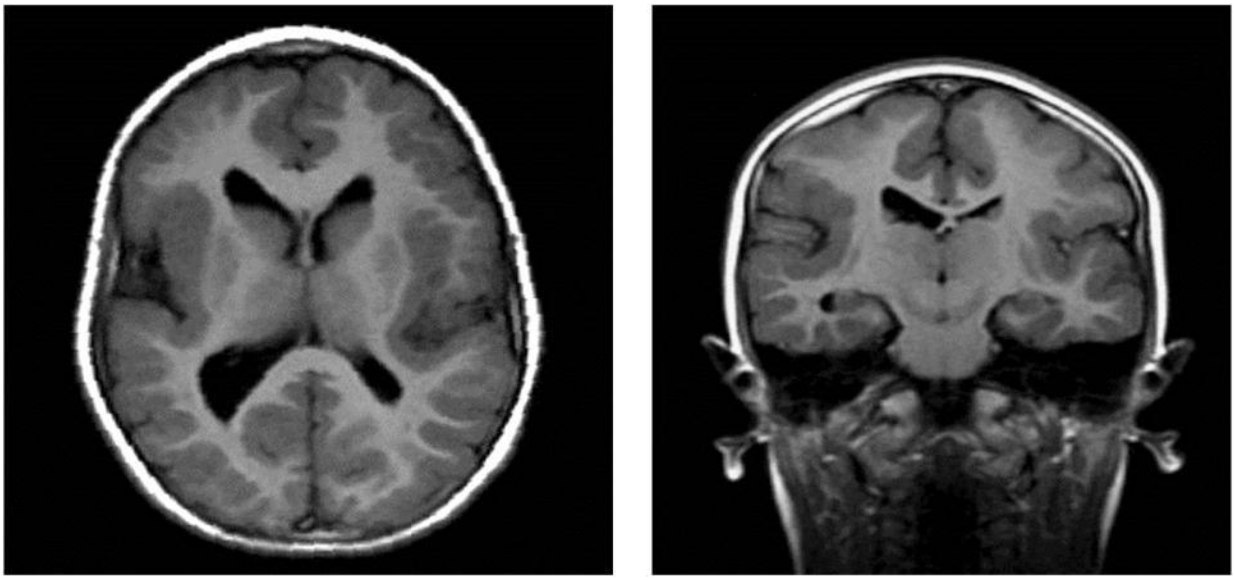

Fig. S19 – MRI scans. Cortical dysplasia.

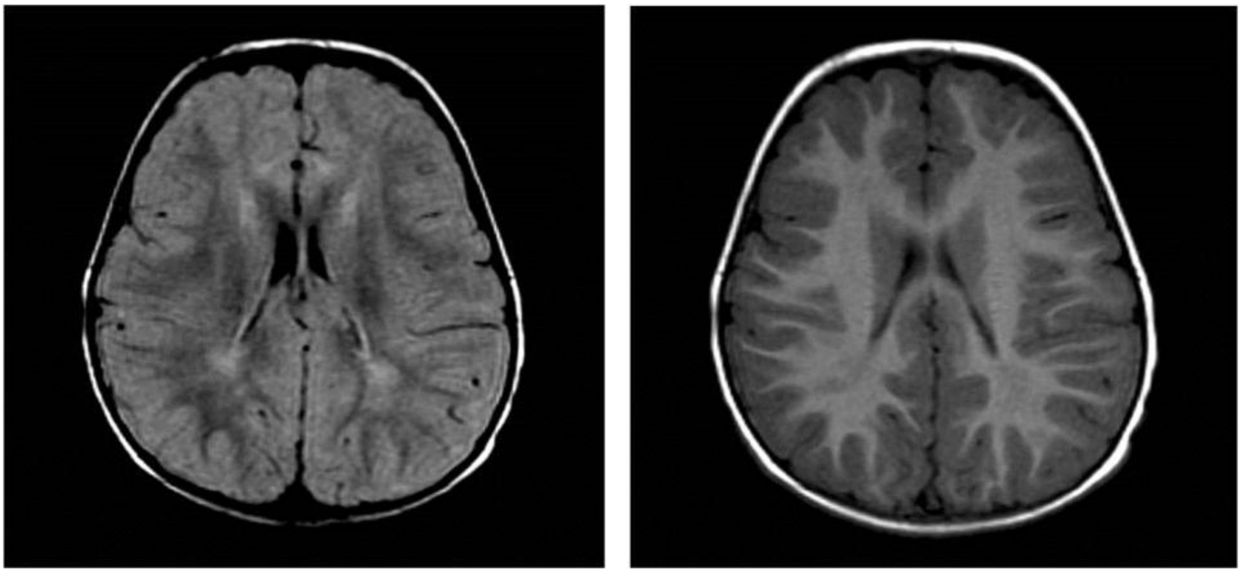

Fig. S20 - MRI scans. Parieto-occipital dysmyelinating areas, characterized by abnormal hyperintense signal on T2-FLAIR image in frontal and parieto-occipital periventricular regions

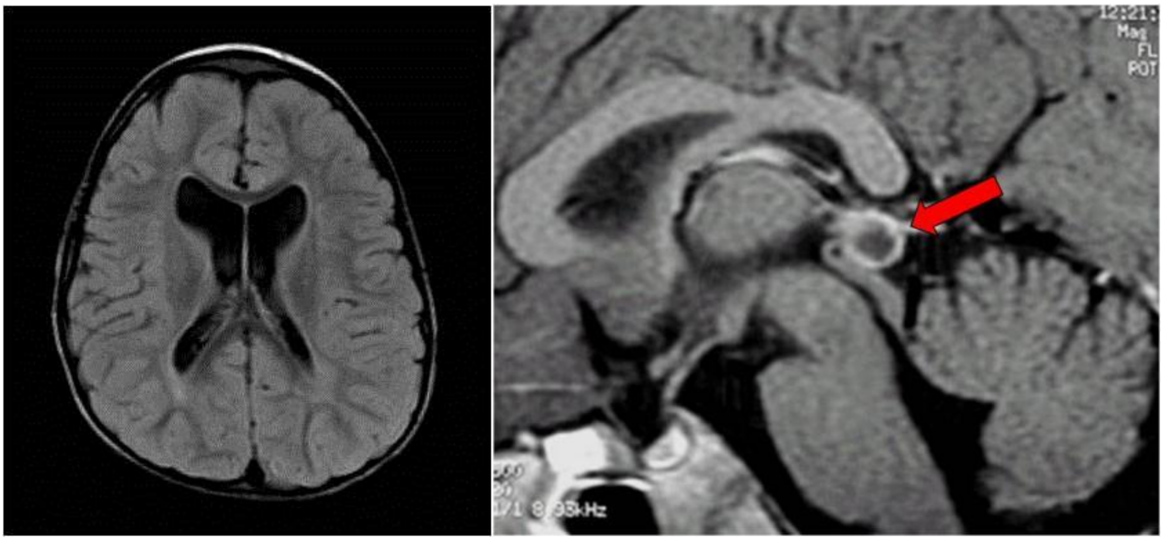

Fig. S21 MRI scans**.** This is an example of periventricular leukomalacia; you can see reduction of the periventricular white matter, abnormal hyperintense signal (on T2-FLAIR image) (of the white matter) in parieto-occipital periventricular regions and “ex vacuo” dilation of the frontal horns. Red arrow: pineal cyst.

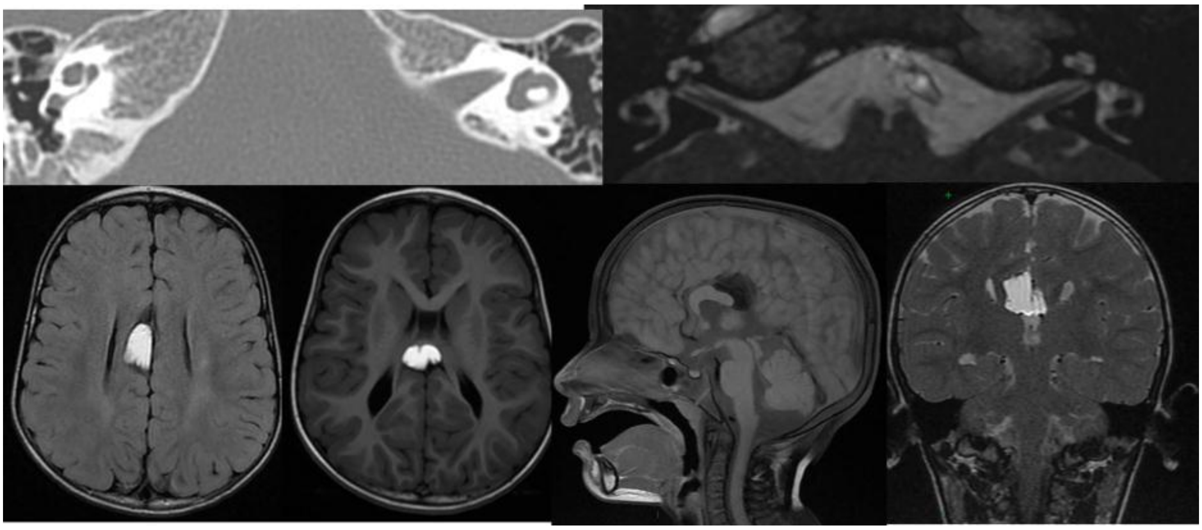

Fig. S22 - CT and MRI scans. Hypoplastic lateral semicircular canal; lypoma of the corpus callosum; agenesis of the posterior part and splenium of the corpus callosum; pellucid septum cyst; dilated cisterna magna (or cerebellomedullary cistern).
